# *MEN1* silencing aggravates tumorigenic potential of AR-independent prostate cancer cells through nuclear translocation and activation of JunD and β-catenin

**DOI:** 10.1186/s13046-021-02058-7

**Published:** 2021-08-26

**Authors:** Yakun Luo, Virginie Vlaeminck-Guillem, Silvère Baron, Sarah Dallel, Chang Xian Zhang, Muriel Le Romancer

**Affiliations:** 1 Université Lyon, Université Claude Bernard Lyon 1, INSERM 1052, CNRS 5286, Centre Léon Bérard, Centre de recherche en cancérologie de Lyon, 69008 Lyon, France; 2 Centre de biologie Sud, Hôpital Lyon Sud, Hospices Civils de Lyon, 69310 Pierre-Bénite, France; 3 Université Clermont Auvergne, GReD, CNRS UMR 6293, INSERM U1103, 28 Place Henri Dunant, BP38, 63001 Clermont-Ferrand, France

**Keywords:** Prostate cancer, AR-independent cells, *MEN1*, JunD, β-Catenin

## Abstract

**Background:**

Recent studies highlighted the increased frequency of AR-low or -negative prostate cancers (PCas) and the importance of AR-independent mechanisms in driving metastatic castration-resistant PCa (mCRPC) development and progression. Several previous studies have highlighted the involvement of the *MEN1* gene in PCa. In the current study, we focused on its role specifically in AR-independent PCa cells.

**Methods:**

Cell tumorigenic features were evaluated by proliferation assay, foci formation, colony formation in soft agar, wound healing assay and xenograft experiments in mice. Quantitative RT-PCR, Western blot and immunostaining were performed to determine the expression of different factors in human PCa lines. Different ChIP-qPCR-based assays were carried out to dissect the action of JunD and β-catenin.

**Results:**

We found that *MEN1* silencing in AR-independent cell lines, DU145 and PC3, resulted in an increase in anchorage independence and cell migration, accompanied by sustained MYC expression. By searching for factors known to positively regulate MYC expression and play a relevant role in PCa development and progression, we uncovered that *MEN1*-KD triggered the nuclear translocation of JunD and β-catenin. ChIP and 3C analyses further demonstrated that *MEN1*-KD led to, on the one hand, augmented binding of JunD to the *MYC* 5′ enhancer and increased formation of loop structure, and on the other hand, increased binding of β-catenin to the *MYC* promoter. Moreover, the expression of several molecular markers of EMT, including E-cadherin, BMI1, Twist1 and HIF-1α, was altered in *MEN1*-KD DU145 and PC3 cells. In addition, analyses using cultured cells and PC3-GFP xenografts in mice demonstrated that JunD and β-catenin are necessary for the altered tumorigenic potential triggered by *MEN1* inactivation in AR-independent PCa cells. Finally, we observed a significant negative clinical correlation between *MEN1* and *CTNNB1* mRNA expression in primary PCa and mCRPC datasets.

**Conclusions:**

Our current work highlights an unrecognized oncosuppressive role for menin specifically in AR-independent PCa cells, through the activation of JunD and β-catenin pathways.

**Supplementary Information:**

The online version contains supplementary material available at 10.1186/s13046-021-02058-7.

## Background

With over 1.4 million new cases and more than 370, 000 deaths reported worldwide in 2020 [1], prostate cancer (PCa) remains a major cause of cancer-related mortality and morbidity in men worldwide. The majority of PCas express the androgen receptor (AR), and dysregulation of the androgen pathway is key to the development and progression of PCa [2]. Androgen deprivation therapies (ADT) are, therefore, a highly effective frontline treatment for PCa [3]. However, ADT are characterized by the virtually unavoidable emergence of resistance, termed castration-resistant PCas (CRPCs), often metastatic (mCRPC) and with a high mortality rate [1, 4]. Genomic characterization of CRPCs has led to their subdivision into two subtypes: (1) AR-dependent CRPCs, containing alterations in the AR gene, such as amplification, point mutations, and generation of splice variants; and (2) AR-independent CRPCs, in which resistant cells lack AR expression or signaling [5]. Markedly, among these AR-independent CRPCs, some PCas express neither the AR nor markers of neuroendocrine (NE) differentiation (“AR null–NE null”, or double negative PCa, DNPC), and their incidence has risen over the past 2 decades from 5% in 1998–2011 to 23% in 2012–2016 [6]. Owing to the heterogeneous nature of the disease, addressing the mechanisms specifically underlying different subtypes of PCa is thus highly relevant.

Studies on DNPC and AR-independent mCRPC have considerably advanced our knowledge in this field over the last decade. By extensively characterizing cellular markers of the related lesions and cell models, cell dedifferentiation and/or altered cell plasticity were proposed to be critical for the development and evolution of these cancers [7]. Among these alterations, epithelial-to-mesenchymal transition (EMT), often in conjunction with stem cell-like changes, is considered to be crucial in the development of AR-negative PCas. The proto-oncogene MYC, a potent transcription factor that controls various biological processes [8–12], was reported to be one of the key drivers of CRPC and neuroendocrine prostate cancer (NEPC) [13–15]. Furthermore, several genetic factors and signaling pathways were shown to bypass AR pathways and thus be involved in AR-independent PCa development, such as P53 [16], RB1 [16], PTEN [17], glucocorticoid receptor (GR) [18], FGF [6] and Stat3/5, as well as the metabolic ACSL pathway [19, 20], whereas the underlying mechanisms remain elusive.

We have previously observed that male heterozygous *Men1* mutant mice developed PCa with a low but significant frequency [21]. However, Malik et al. reported that the physical interaction between menin and AR was essential for the growth of human PCa cell lines both in culture and in xenografts [22]. Recently, we demonstrated the decisive role played by the menin protein, encoded by the *MEN1* gene, in regulating *AR* transcription in AR-dependent PCa cell lines [23]. Our findings also highlighted disparities in the effects of this protein on the proliferation of PCa cells, since *MEN1* silencing had no impact (PC3 cells), or even an increased (DU145 cells) proliferation of AR-independent cells, whereas it led to a marked decrease in cell growth of AR-dependent lines (LNCaP, 22Rv1 and VCaP). The distinct role of the *MEN1* gene in these two PCa cell populations, albeit intriguing, is not surprising, considering the different cellular and molecular mechanisms involved in their tumorigenesis. Indeed, the *MEN1* gene is largely known as a tumor suppressor in several types of endocrine tissues, since its mutation predisposes patients to multiple endocrine neoplasia type 1 syndrome (MEN1 syndrome, OMIM 131100). However, it is now well established that menin displays oncogenic effects in certain types of leukemia containing fusion MLL caused by chromosome translocation [24]. Dreijerink et al. found that the gene expression profile obtained after *MEN1* silencing in normal mammary luminal progenitors was highly distinct from that found in ER-positive breast cancer MCF7 cells, suggesting that menin regulates different gene sets in normal mammary luminal cells versus ER-positive breast cancer cells [25]. Here, we focused on the mechanisms underlying the distinct effects observed upon *MEN1* silencing in AR-independent PCa cells. Through different analyses using cultured cells and in vivo experiments, our data unveiled the activation of the JunD and β-catenin pathways upon menin inactivation in AR-independent PCa cells, underscoring menin as an oncosuppressive factor in these cells, in marked contrast to its role in AR-dependent PCa cells.

## Materials and methods

### Cell culture and treatment

Prostate cancer cell lines were purchased from ATCC. Their authentication was renewed recently and *Mycoplasma* testing was carried out regularly. LNCaP, 22Rv1 and DU145 were cultured in RPMI medium, and PC3 cells in F-12 medium (Gibco Invitrogen), at 37 °C with 5% CO_2_. Inhibition of menin-MLL interaction was achieved through the use of MI503 (Active Biochem).

### Cell proliferation and foci formation assays

Cell proliferation assays were performed as described previously [23]. For foci formation assays, cells were seeded in 6-well culture plates at 5 × 10^2^ cells for LNCaP, and at 2.5 × 10^2^ cells for 22Rv1, DU145 or PC3. Cells were transfected with siRNA or treated with MI503, and cultured for 2 weeks. The ensuing colonies were stained with 0.05% crystal violet. The images of the plates were analyzed using Image J software. Each experiment was conducted in triplicate and statistical analyses were performed using the Prism software.

### Scratch wound healing assay

Cell migration capacities were evaluated through wound healing assays. 5 × 10^3^ cells were seeded onto 6-well plates. After 24 h in culture, cells were transfected with 20 nM of siRNA. 72 h after transfection, wounds were created, and wound closures were captured at 0, 6, and 12 h.

### Colony formation in soft agar

Soft agar assays were performed as described previously [26]. After 4 weeks of incubation at 37 °C, colonies were stained with 0.05% (w/v) crystal violet (Sigma) and colonies were counted using Image J software.

### *X*enograft tumor growth test

Xenografts were performed using 8-week-old male NOD-Scidγ mice by surgical implantation under the kidney capsule. 1 × 10^5^ PC3-GFP cells were encapsulated in a collagen matrix as previously described [27]. Five days after implantation, mice were randomized into two groups and treated three-times a week for 1 month. Mouse groups were treated with i.p. injections of menin inhibitor MI503 75 mg/kg (MedChemTronica) (*n* = 11) or Vehicle solution (DMSO/PEG300, Sigma-Aldrich) (*n* = 9). One month after, xenografted tissues were collected after necropsy and processed for further analyses. The quantification of the number of PC3-GFP cells in xenografted tissues by qPCR assay was performed as previously described [28]. All experiments were approved by Auvergne Ethics Committee (CEMEAA) and registered according the approval number 17296–2018102216428025 v3.

### RNA interference and transfection

Transfection of siRNA was performed using Lipofectamine 2000 (Invitrogen) according to the manufacturer’s instructions, and incubated for 72 h.

### RNA extraction, reverse transcription, and real-time PCR

Total RNA from cultured cells was extracted using the RNeasy-Kits (Qiagen, Valencia, USA) as per manufacturer’s instructions. cDNAs were amplified and quantified in an ABI Prism 7500 Sequence Detection System (Applied Biosystems) using the SYBR Green I dye (SsoAdvanced Universal SYBR Green, Bio-Rad). Data were normalized against the in-house control HPRT and represented as fold change. Primers used are listed in the [Media MOESM1].

### Protein extraction and Western blotting

Subcellular fractions were separated using NE-PER Nuclear and Cytoplasmic Extraction Reagents (ref. 78,833, Thermo Scientific). Western blotting was carried out according to the method described previously [29].

### Immunofluorescence (IF) and immunohistochemistry (IHC) staining

For IF staining, cells were grown on glass coverslips, then fixed with methanol for 5 min at room temperature. Following fixation, cells were blocked with Dako buffer (S0809, Agilent) for 1 h, and incubated with primary antibodies overnight at 4 °C, then with appropriate secondary antibodies conjugated with Alexa 555 (red) or Alexa 488 (green) (Cell Signaling Technology). Cells were counterstained with DAPI (DUO82040, Sigma-Aldrich) for 10 min and visualized by fluorescence microscopy (Eclipse-NiE NIKON microscope).

Xenografts were collected and fixed in 4% PFA prior to paraffin embedding, sectioning, staining with hematoxylin and eosin, and with immunostaining conducted as described previously [23]. Images were acquired on a ZEISS Axioscope 5 microscope.

### Chromatin immunoprecipitation (ChIP) and sequential ChIP (reChIP) assays

Chromatin immunoprecipitation (ChIP) was performed with the Millipore ChIP Assay Kit (17–295) as described previously [23]. Briefly, cells were crosslinked with 1% formaldehyde for 10 min at 37 °C. Chromatin was prepared according to the Millipore protocol and sonicated to an average size of 300–500 bp using a Diagenode Bioruptor. Chromatin fragments were immunoprecipitated at 4 °C overnight with menin antibody or normal rabbit IgG used as a negative control (see references of antibodies in the [Media MOESM1]), and immune complexes were collected on Protein A agarose beads (ChIP assay kit, 17–295, Millipore).

For reChIP assays, the first immunoprecipitated chromatin complexes were washed and eluted with 10 mM dithiothreitol at 37 °C for 30 min and diluted 50-fold with ChIP dilution buffer. The second immunoprecipitations were then performed [30]. Each ChIP or reChIP assay was repeated at least three times independently. Primers used for ChIP-qPCR are listed in the [Media MOESM1].

### Chromosome conformation capture (3C) assay and ChIP-3C

3C and ChIP-3C assays were performed as described previously [31–33]. A comprehensive description of all the process of experiment, reagents, kits, antibodies, and primers used in this study can be found in [Media MOESM1].

### Raw data for mining analysis

The cBioPortal for cancer genomics (http://www.cbioportal.org) [34] was employed to analyze mRNA co-expression between *MEN1* and *JunD*, *MEN1* and *CTNNB1* in PCa. Data on the correlation between *MEN1* and *JunD* mRNA expression were download from SU2C/PCF Dream team (mCRPC) [35] and Firehose Legacy team and PanCancer Altas team (Primary prostate adenocarcinoma) [36].

### Statistical analysis

All experiments were repeated at least three times to ensure accuracy. The values are expressed as mean ± standard deviation (S.D.) and analyzed by *Student t-test*. The level of significance: ns, non-significant vs Ctrl, *P < 0.05, ** *P* < 0.01, *** *P* < 0.001, for all analyses.

## Results

### *MEN1* silencing promotes the tumorigenic potential and maintains MYC expression in AR-independent PCa cells

**Fig. 1:**
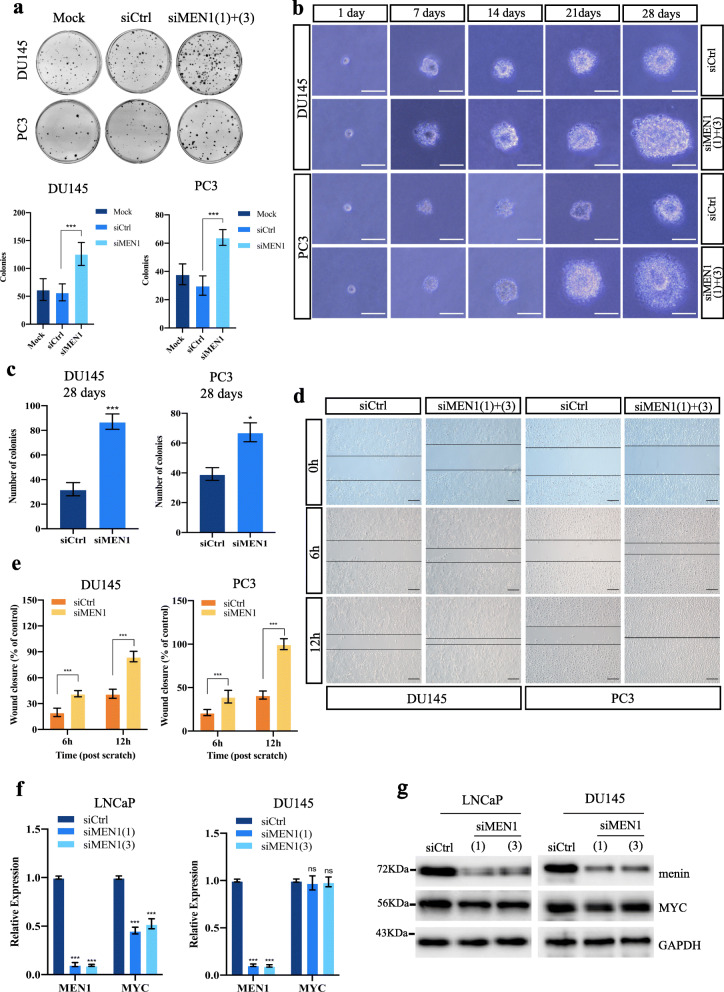
*MEN1* silencing promotes anchorage independence and cell migration in AR-independent PCa cells with maintained MYC expression. **a** Upper panel: Representative images of foci formation assay with DU145 and PC3 cells treated with siMEN1(1) + (3) or siCrtl. Lower panel: Quantification of foci formation assay. **b** Representative images of soft agar colony formation assay in MEN1-KD DU145 or PC3 cells. Scale bar = 50 μm. **c** Bar charts showing colony formation 28 days post-transfection with siMEN1 or siCtrl, extrapolated from images using Image J software. **d** Representative images of scratch wound healing assays using siMEN1 or siCtrl-transfected DU145 and PC3 cells. Scale bar = 200 μm. **e** Graphs indicating cell migration displayed in terms of the % wound closure 6 h and 12 h post-wounding (t = 0, as control). **f** and **g** Quantitative RT-PCR (qRT-PCR) analysis of MYC transcripts (**f**) and Western blot analysis of MYC protein levels (**g**) in PCa cells treated with siCtrl or siMEN1(1) + (3) for 72 h. Representative blots were as performed of three independent experiments

Our previous results, showing that *MEN1* silencing had distinct effects on cell proliferation of AR-dependent versus AR-independent PCa cells [23], prompted us to dissect cellular consequences of *MEN1* inactivation in AR-independent PCa cells. Firstly, we performed foci formation and soft agar assays to assess cell tumorigenic potential in *MEN1***-**knockdown (KD) PCa cells. *MEN1*-KD resulted in a significant increase in colony formation in AR-independent PCa cells (DU145 and PC3) in both tests (Fig. 1a-b), whereas it had the opposite effect in AR-dependent PCa cells (LNCaP and 22Rv1) tested for foci formation (Fig. S1a-b). Interestingly, *MEN1*-KD also led to a decreased number of colonies formed in PC3 cells re-expressing AR (PC3-AR), to a lesser extent than in AR-dependent cells but significantly greater than AR-independent PCa cells (Fig. S1a-b), consistent with the previously observed reduced cell proliferation in *MEN1*-KD PC3-AR cells [23]. The efficiency of *MEN1* silencing was confirmed by Western blotting (Fig. S1b). In soft agar assays, *MEN1*-KD DU145 and PC3 cells gave rise not only to more foci, but also to larger ones, with colonies appearing more irregular in shape, compared with siCtrl-treated cells (Fig. 1b). Furthermore, to determine whether menin influenced cell migration, *MEN1*-KD DU145 and PC3 cells were subjected to scratch wound-healing assays, with wound closure being monitored at 6 h and 12 h. A significant increasing in DU145 and PC3 cell migration was observed at these time-points following *MEN1* silencing with siMEN1(1) + (3) (Fig. 1c-d), whereas this reduced cell migration of 22Rv1 and PC3-AR cells (Fig. S1c). Taken together, *MEN1*-KD in DU145 and PC3 cells promoted cell growth in an anchorage-independent manner and increased cell migration, suggesting that menin plays a tumor suppressive role in AR-independent PCa cell lines, unlike its oncogenic role in AR-dependent PCa cell lines.

Intriguingly, Wu et al. demonstrated that menin physically interacts with MYC to enhance the transcription of MYC target genes in liver cancer cells [37]. We observed that, while *MEN1*-KD reduced the expression of MYC at the transcriptional and protein levels in LNCaP and 22Rv1 cells, its expression was maintained in the two *MEN1*-KD AR-independent PCa cells (Fig. 1f-g and Fig. S1d), suggesting that the difference in cellular activity between AR-dependent and AR-independent PCa cells could be, at least partially, due to MYC expression. Having shown that *MEN1*-KD increased the tumorigenic potential of AR-independent PCa cells and that MYC expression was maintained, we wondered whether factors known to regulate MYC expression could contribute to the effects triggered by *MEN1* silencing.

### Reduced menin expression triggers the nuclear translocation of JunD in AR-independent PCa cells

**Fig. 2:**
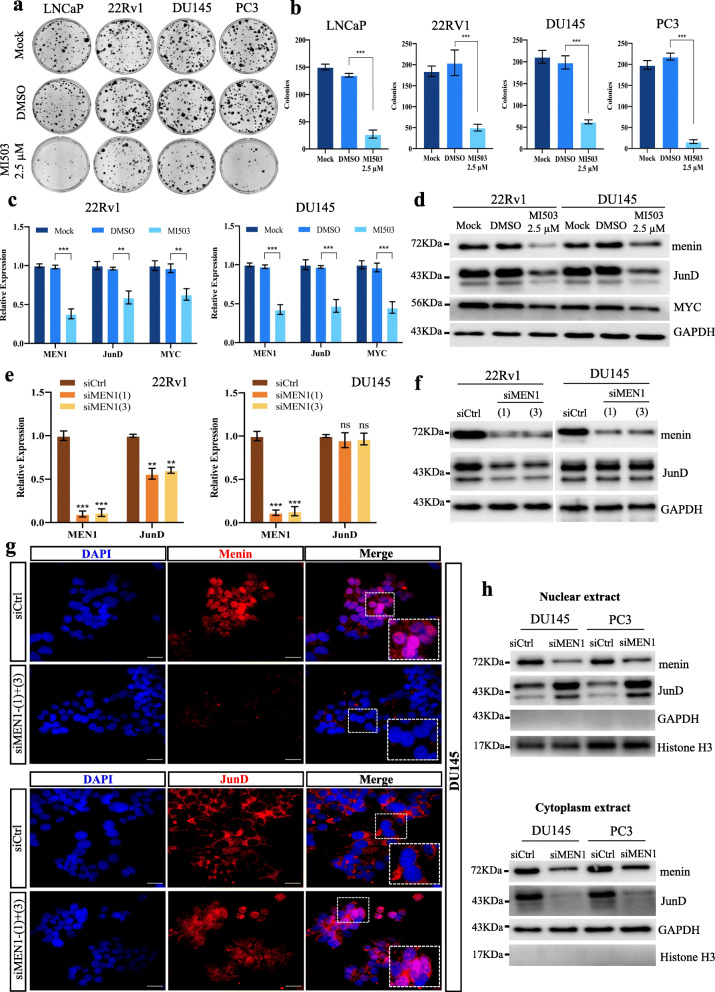
*MEN1* silencing triggers the nuclear translocation of JunD in AR-independent PCa cells. Representative images of foci formation assays (**a**) and their quantification (**b**) using LNCaP, 22Rv1, DU145 and PC3 cells treated or not with MI503 (2.5 μM). qRT-PCR analysis of *JunD* and *MYC* mRNA expression (**c**) and Western blot analysis showing JunD and MYC expression (**d**) in 22Rv1 and DU145 cells treated or not with MI503 (2.5 μM). qRT-PCR analysis of *JunD* mRNA expression (**e)** and Western blot analysis of JunD protein expression (**f**) in *MEN1*-KD 22Rv1 and DU145 cells. **g** IF staining showing menin and JunD in DU145 cells treated with siCtrl or siMEN1(1) + (3). Scale bar = 25 μm. **h** Western blot analysis of JunD expression in different subcellular fractions in DU145 and PC3 cells treated with siCtrl or siMEN1(1) + (3). Representative blots of three independent experiments

One factor in particular, the proto-oncogene JunD, was recently reported to promote the proliferation of PCa cells through MYC signaling [8]. To better understand this mechanism, we investigated the distinct effects of *MEN1*-KD in AR-dependent and -independent PCa cells using MI503 as a means of comparison, since MI503 is known to inhibit not only the interaction between menin and MLL, but also that of menin and JunD [38]. Indeed, we previously demonstrated that MI503 treatment inhibited the proliferation of both types of PCa cell lines, unlike siMEN1 treatment [23]. Hence, we initially performed foci formation assays to confirm a significant decrease in colony formation in both AR-dependent and AR-independent PCa cells upon MI503 treatment (Fig. 2a-b). To understand the molecular differences triggered by siMEN1 and MI503, we examined the expression of MYC and JunD. The expression of *MEN1*, *MYC* and *JunD* was lower in all MI503-treated PCa cells at the mRNA (Fig. 2c and Fig. S2a) and protein levels (Fig. 2d). However, following *MEN1*-KD, only AR-dependent (LNCaP and 22Rv1) cells displayed a decrease in mRNA (Fig. 2e and S2b) and protein levels (Fig. 2f and S2c). Indeed, AR-independent PCa cells remained unaffected by this treatment (Fig. 2e-f and S2b-c), indicating that MI503 suppresses cell growth by inhibiting menin and JunD expression in PCa cells. Consequently, we confirmed the positive role of JunD on cell proliferation in *JunD*-KD DU145 and PC3 cells (Fig. S2d). We further evaluated JunD protein expression in *MEN1*-KD DU145 and PC3 cells, and found that, although *MEN1* silencing did not affect the JunD expression at the transcriptional (Fig. 2e and S2b, lower panel) and total protein levels (Fig. 2f and S2c), it triggered the nuclear translocation of JunD in these AR-independent PCa cells (Fig. 2g-h and S2e), indicative of JunD activation [39]. Conversely, *JunD* silencing did not lead to any change in menin expression (Fig. S2f-g).

### *MEN1* knockdown enhances the binding of JunD to the *MYC* locus in AR-independent PCa cells

**Fig. 3:**
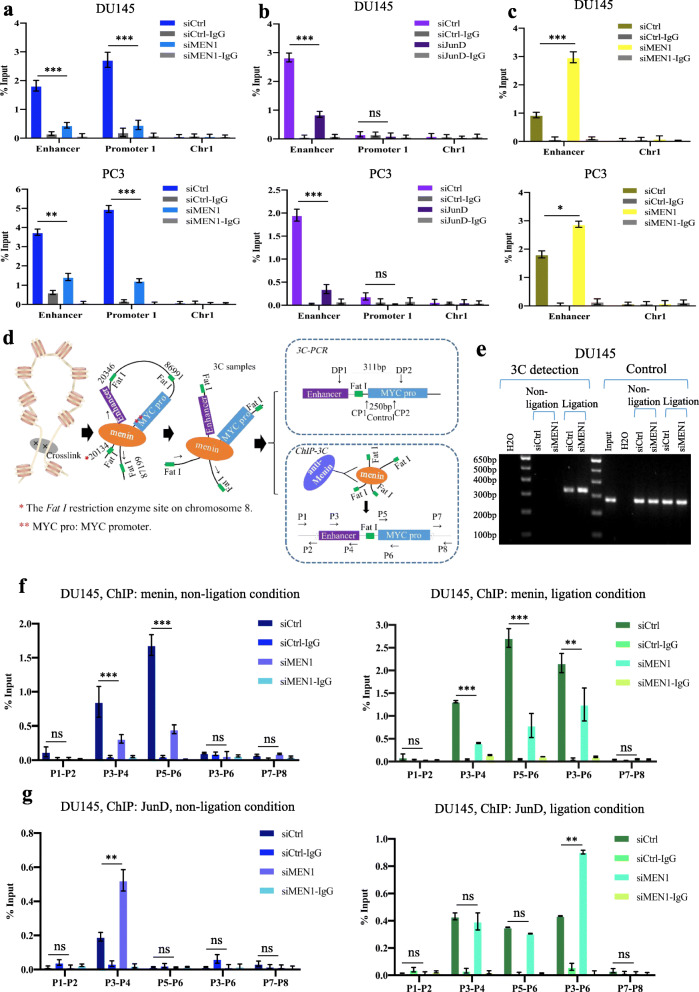
*MEN1* knockdown leads to increased *JunD* binding and loop structure formation in the MYC locus in AR-independent *PCa* cells. **a** ChIP-qPCR analysis using anti-menin to evaluate the binding of menin to the *MYC* 5′ enhancer (− 67 kb) and promoter in DU145 and PC3 cells treated with siCtrl or siMEN1(1) + (3). *Chr1* served as a negative control. **b** ChIP-qPCR analysis using anti-JunD to detect the binding of JunD to the *MYC* 5′ enhancer and promoter in DU145 and PC3 cells treated with siCtrl or siJunD. **c** ChIP-qPCR analysis to assess the effect of *MEN1*-KD on the level of JunD recruitment to the *MYC* 5′ enhancer in DU145 and PC3 cells. **d** Primer locations for qPCR of 3C and ChIP-3C analyses. **e** 3C detection results (DP1-DP2 fragment) and control (CP1-CP2) by PCR in siCtrl or siMEN1(1) + (3)-transfected DU145 cells upon ligation or non-ligation. ChIP-3C qPCR analysis detecting menin ChIP-qPCR analysis showing menin binding (**f)** and JunD binding (**g**) to the *MYC* 5′ enhancer, *MYC* promoter and the looping fragment (F3-F6) in DU145 cells transfected with siCtrl or siMEN1(1) + (3) under ligation (right panel) or non-ligation (left panel) conditions, P1-P2 and P7-P8 were used as negative controls. Representative blots of three independent experiments

Wang et al. identified four potential AP-1 binding sites in a 5′ *MYC* enhancer, situated 67 kb upstream of the transcription start site (TSS) of *MYC*, and demonstrated the binding of JunD to the enhancer to regulate *MYC* transcription in breast cancer cells [40]. We wondered whether the activation of JunD may allow the maintenance of *MYC* transcription in *MEN1*-KD AR-independent PCa cells. Through ChIP-qPCR analyses, we observed that menin bound to the *MYC* promoter and to its 5′ enhancer in DU145 and PC3 cells (Fig. 3a), while JunD bound only to the *MYC* 5′ enhancer (Fig. 3b). We then hypothesized that the increased JunD nuclear translocation triggered by *MEN1* silencing may augment its binding to the 5′ *MYC* enhancer, which we confirmed by ChIP analysis upon *MEN1*-KD in DU145 and PC3 cells (Fig. 3c).

Next, we performed 3C assays to determine whether reduced menin expression modulates the “loop” structure between the distal enhancer region and the proximal promoter region [40]. For the 3C-PCR reaction, we amplified a 330 bp DNA fragment (“Detection fragment”) to assess intramolecular ligation of the looping between the enhancer and promoter of the *MYC* locus, using the forward primer (DP1) that anneals upstream of the distal enhancer and the reverse primer (DP2) that anneals downstream from the *Fat I* site in the *MYC* promoter (Fig. 3d). As shown in Fig. 3e and Fig. S3a, the detection fragment increased in *MEN1*-KD cells under the ligation condition, compared to siCtrl cells. As expected, the detection fragment failed to yield products in 3C assays under the non-ligation condition. This analysis clearly demonstrates that reduced menin expression triggers increased loop formation between the *MYC* enhancer and proximal promoter in DU145 and PC3 cells.

We then performed ChIP-3C [41] to further determine how the loop formed between the *MYC* enhancer and proximal promoter [42] might change in *MEN1*-KD AR-independent PCa cells. In the non-ligation condition, menin bound to the enhancer and promoter of the *MYC* locus (Fig. 3f and S[Media MOESM2]b, left panels), and JunD bound only to the 5′ *MYC* enhancer in DU145 and PC3 cells (Fig. 3g and S[Media MOESM2]c, left panels), while, in the ligation condition, both menin and JunD could be detected on the 5′ *MYC* enhancer and proximal promoter, as well as on the intramolecular ligation region (P3-P6), suggesting that they bound together to the looping structure (Fig. 3f-g and S[Media MOESM2]b-c, right panels). Interestingly, JunD binding increased at the intramolecular ligation region (P3-P6) of the *MYC* locus in *MEN1*-KD DU145 and PC3 cells under the ligation condition (Fig. 3g and S[Media MOESM2]c, right panels). These results provide first evidence that menin is present within the corresponding chromatin loop structure between the *MYC* enhancer and promoter, and that, importantly, its inactivation leads to increased binding of JunD to the 5′ *MYC* enhancer and the loop structure. Taken together, our analyses strongly suggest that JunD, through its nuclear translocation, replaces menin on the *MYC* locus to maintain *MYC* transcription in *MEN1*-KD AR-independent PCa cells.

### *MEN1* silencing triggers the nuclear translocation of β-catenin in AR-independent PCa cells

**Fig. 4:**
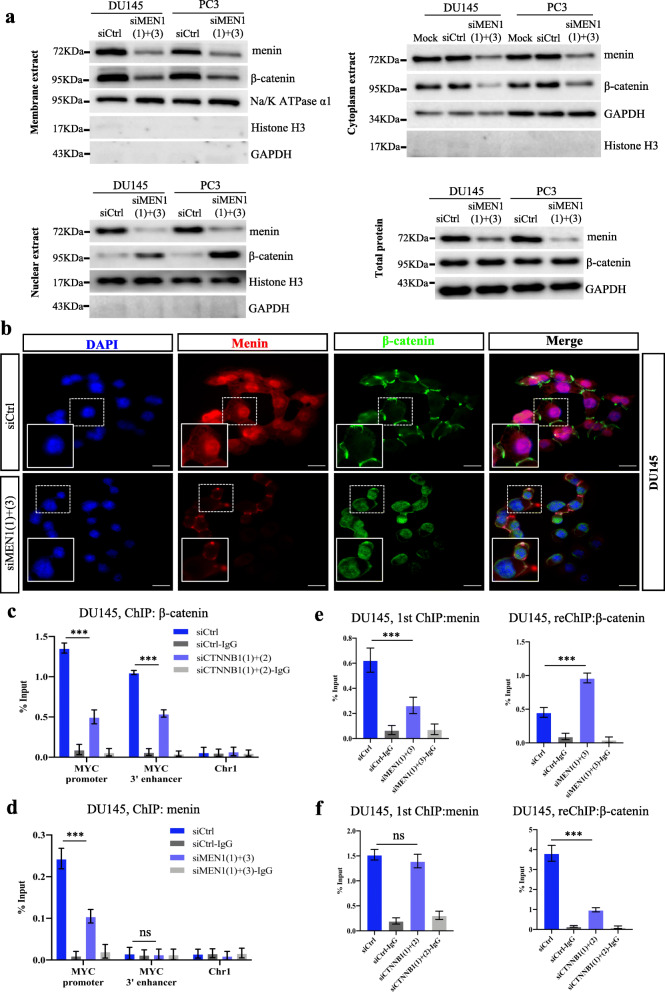
*MEN1* silencing results in nuclear translocation of β-catenin and an increase in its binding to the MYC promoter in AR-independent *PCa* cells. **a** Western blot analysis of β-catenin expression in different subcellular fractions in siCtrl or siMEN1(1) + (3)-treated DU145 and PC3 cells as indicated. **b** Double IF staining showing menin and β-catenin in siCtrl or siMEN1(1) + (3)-treated DU145 cells. Scale bar = 25 μm. ChIP-qPCR analysis assessing β-catenin (**c**) or menin (**d**) binding to the MYC promoter and the MYC 3’enhancer in siCtrl or siMEN1(1) + (3)-treated DU145 cells. reChIP analysis evaluating the effect of MEN1-KD (**e**) and CTNNB1-KD (**f**) on menin (left panel) and β-catenin (right panel) co-occupancy on the MYC promoter in DU145 cells. Representative blots of three independent experiments

Our previous works highlighted the activation of β-catenin, a menin-interacting protein, upon *Men1* disruption in mouse insulinoma [43, 44]. Moreover, β-catenin is a well-known oncogene in prostate cancer cells [45]. We, therefore, also analyzed the expression of β-catenin in *MEN1*-KD PCa cells, and found that *MEN1* silencing resulted in the accumulation of β-catenin in the nucleus of DU145 and PC3 cells, with its membrane and cytoplasmic expression being markedly reduced (Fig. 4a-b and Fig. S4a). Conversely, *MEN1*-KD significantly downregulated β-catenin total protein and cytoplasmic fraction levels in LNCaP cells and 22Rv1 cells, whereas its expression in nuclear and membrane subcellular fractions remained unaltered (Fig. S[Media MOESM2]b-d). However, *CTNNB1* silencing did not result in any change in menin expression (Fig. S[Media MOESM2]e). Furthermore, we confirmed the positive role of β-catenin in cell proliferation in DU145 and PC3 cells knocked-down for *CTNNB1* coding for β-catenin (Fig. S[Media MOESM2]f). Collectively, these results indicate that, in *MEN1*-KD AR-independent PCa cells, the nuclear translocation of β-catenin is drastically increased, a hallmark of the activation of the WNT signaling pathway [46].

### *MEN1* inactivation increases β-catenin binding to the *MYC* promoter in AR-independent PCa cells

It has previously been reported that MYC and β-catenin have a strong cooperative action in different cancers [47–49]. We thus investigated the eventual interplay between menin and β-catenin in regulating MYC expression in AR-independent PCa cells. Our ChIP analyses showed that β-catenin bound to the *MYC* promoter and its 3′ enhancer, formerly described in colon cancer cells [48] (Fig. 4c and S[Media MOESM2]a), while menin bound to the promoter, but not to the *MYC* 3′ enhancer in DU145 and PC3 cells (Fig. 4d and S[Media MOESM2]b). Furthermore, ChIP-reChIP and ChIP analyses showed that *MEN1*-KD enhanced β-catenin binding to the *MYC* promoter (Fig. 4e and S[Media MOESM2]c), but not its binding to the *MYC* 3′ enhancer (Fig. S[Media MOESM2]d) in DU145 and PC3 cells, whereas *CCTNB1*-KD did not affect the binding of menin to the *MYC* promoter in DU145 and PC3 cells (Fig. 4f and S[Media MOESM2]e). These analyses indicate that nuclear translocation of β-catenin allows the maintenance of *MYC* transcription in *MEN1*-KD AR-independent PCa cells.

### JunD and β-catenin are critical for the tumorigenic potential of AR-independent PCa cells and the expression of EMT markers

**Fig. 5:**
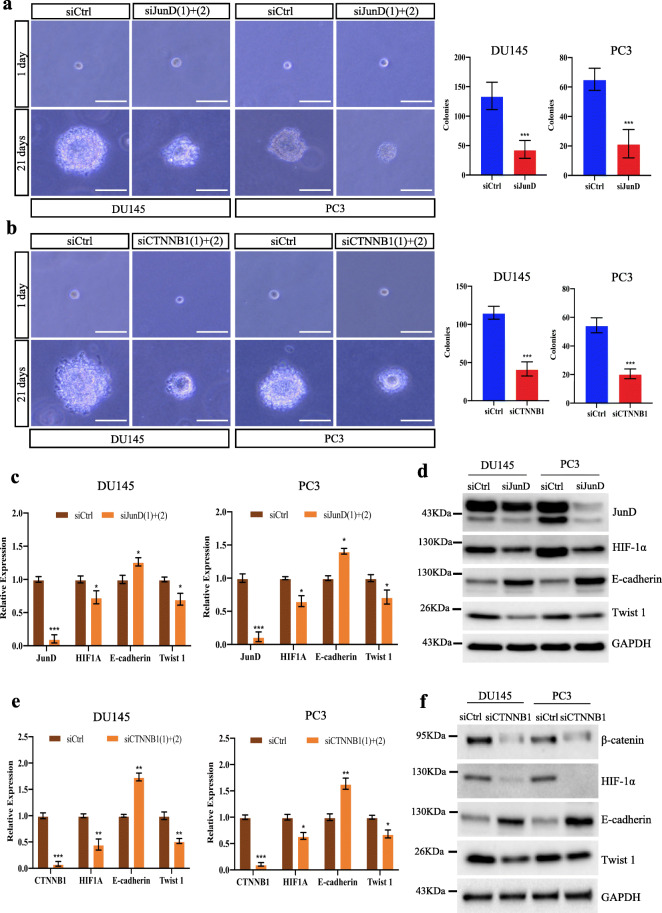
*JunD* and β-catenin are involved in the regulation of EMT marker expression in AR-*independent PCa* cells. Representative images of soft agar colony formation assay in JunD-KD (**a**, left panel) and CTNNB1-KD (**b**, left panel) DU145 or PC3 cells. Scale bar = 50 μm. Graphs showing quantitative analysis of colony formation at 21 days post-transfection with siCtrl or siJunD(1) + (2) using Image J software (right panel). qRT-PCR (**c**) and Western blot (**d**) analyses of mRNA and protein expression of HIF-1α, E-cadherin and Twist 1 in JunD-KD DU145 and PC3 cells. qRT-PCR (**e**) and Western blot (**f**) analyses of mRNA and protein expression of HIF-1α, E-cadherin and Twist 1 in CTNNB1-KD DU145 and PC3 cells DU145 and PC3 cells. Representative blots of three independent experiments

To further investigate the role of JunD and β-catenin in AR-independent PCa cells, we initially performed soft agar assays to assess the anchorage independence in *JunD*-KD or *CTNNB1*-KD PCa cells. Both *JunD*-KD (Fig. 5a) and CTNNB1-KD (Fig. 5b) resulted in a significant decrease in colony formation in DU145 and PC3 cells. Concomitantly, *JunD*-KD (Fig. 5c-d) and *CTNNB1*-KD (Fig. 5e-f) upregulated mRNA and protein levels of the epithelial marker E-cadherin in DU145 and PC3 cells. Consistently, a marked decreased in mRNA and protein expression of Twist 1, a repressor of E-cadherin gene transcription and a known regulator of EMT [50], was observed. Importantly, the expression of HIF-1α, a key mediator in EMT, inflammation and tumorigenesis under hypoxic conditions [51–53], was also reduced in *JunD*-KD and *CTNNB1*-KD DU145 and PC3 cells. These findings indicate that JunD and β-catenin are critical for the tumorigenic potential and the expression of EMT markers in AR-independent PCa cells.

### The activation of JunD and β-catenin is needed to reverse the oncosuppressive role of menin in AR-independent PCa cells

**Fig. 6:**
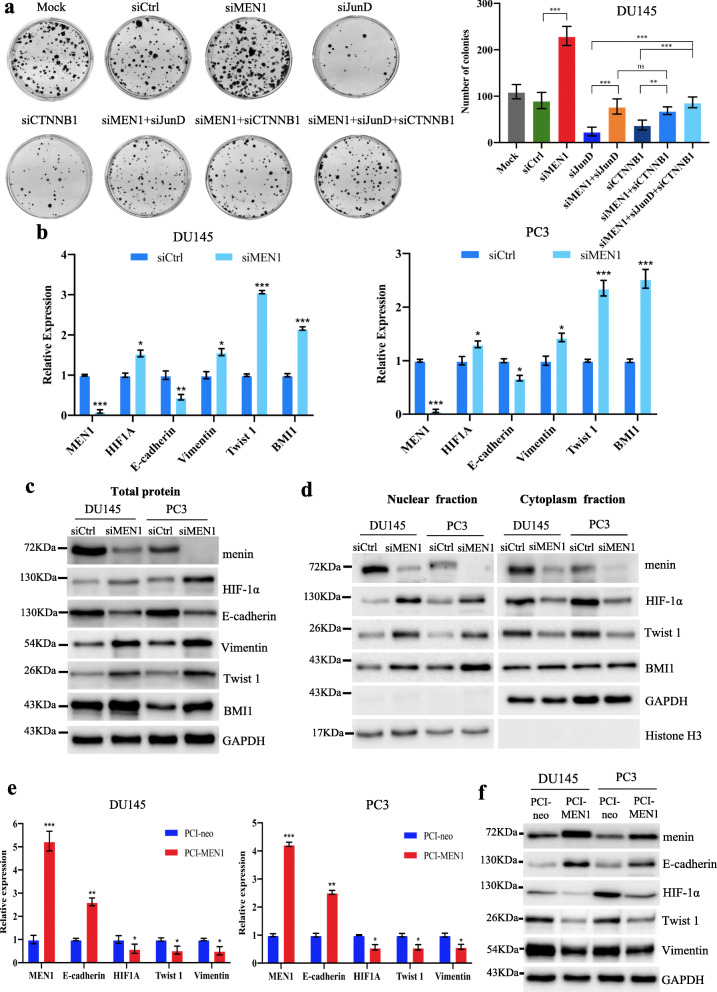
The expression of both *JunD* and β-catenin is critical for the tumorigenic potential of AR-independent *PCa* cells. **a** Representative images of foci formation of DU145 cells upon transfection with siMEN1, siJunD, siCTNNB1, siMEN1 + siJunD, siMEN1+ siCTNNB1 or siMEN1 + siJunD+siCTNNB1. Quantification of data is shown in the right and lower panel. **b** qRT-PCR analysis showing mRNA expression of *HIF-1α, E-cadherin, Vimentin, Twist 1* and *BMI1* in DU145 and PC3 cells transfected with siCtrl or siMEN1(1) + (3). **c** Western blot analysis showing total protein expression of menin, HIF-1α, E-cadherin, Vimentin, Twist 1 and BMI1 in DU145 and PC3 cells transfected with siCtrl or siMEN1(1) + (3). **d** Western blot analysis showing menin, HIF-1α, Vimentin, Twist 1 and BMI1 expression in nuclear and cytoplasmic subcellular fractions in siCtrl or siMEN1(1) + (3)-treated DU145 and PC3 cells. Representative blots of three independent experiments. **e** qRT-PCR analysis of mRNA expression of *E-cadherin, HIF-1α, Twist 1* and *Vimentin* in PCI-neo or PCI-MEN1-transfected DU145 and PC3 cells. **f** Western blot analysis of E-cadherin, HIF-1α, Twist 1 and Vimentin protein expression in DU145 and PC3 cells transfected with PCI-neo or PCI-MEN1 as indicated. Representative blots of three independent experiments

We then proceeded to further determine the specific roles of JunD and β-catenin in *MEN1*-KD AR-independent PCa cells. To this end, we used the foci formation assay to evaluate cell growth upon transfection with siMEN1, siJunD, siCTNNB1, siMEN1 + siJunD, siMEN1 + siCTNNB1 or siMEN1 + siJunD+siCTNNB1 in DU145 (Fig. 6a) and PC3 (Fig. S[Media MOESM2]a) cells. Knockdown of *JunD* or *CTNNB1* significantly abolished the effect of *MEN1*-KD, whereas reduced menin expression significantly reversed the effects of *JunD*-KD or *CTNNB1*-KD in DU145 and PC3 cells (Fig. 6a and S[Media MOESM2]a). We obtained similar results on cell proliferation by Incucyte ZOOM analysis (Fig. S[Media MOESM2]b).

Having shown that both JunD and β-catenin activation are crucial for cell growth in *MEN1*-KD DU145 and PC3 cells, we hypothesized that *MEN1* silencing also alters the expression of genes promoting EMT in AR-independent PCa cell lines. As expected, *MEN1*-KD in DU145 and PC3 cells led to reduced E-cadherin expression at the mRNA and total protein levels (Fig. 6c-d). Moreover, this silencing led to an increase in the expression of HIF-1α, Vimentin and BMI1 at the transcript and total protein levels in these cells, as well as the nuclear accumulation of HIF-1α, Twist 1 and BMI1 (Fig. 6c-d). These results suggest that menin could be a key factor inhibiting the molecular program favoring EMT in AR-independent PCa cells.

Having detected the increased tumorigenic potential and altered expression of several known EMT and stemness markers in *MEN1*-KD AR-independent cells, we overexpressed menin in these cells, in order to further validate our observations. Interestingly, menin overexpression in these cells led to reduced colony formation and cell migration (Fig. S[Media MOESM2]a-b), and increased expression of E-cadherin and reduced expression of Vimentin, HIF-1α and Twist1 (Fig. 6e-f).

**Fig. 7:**
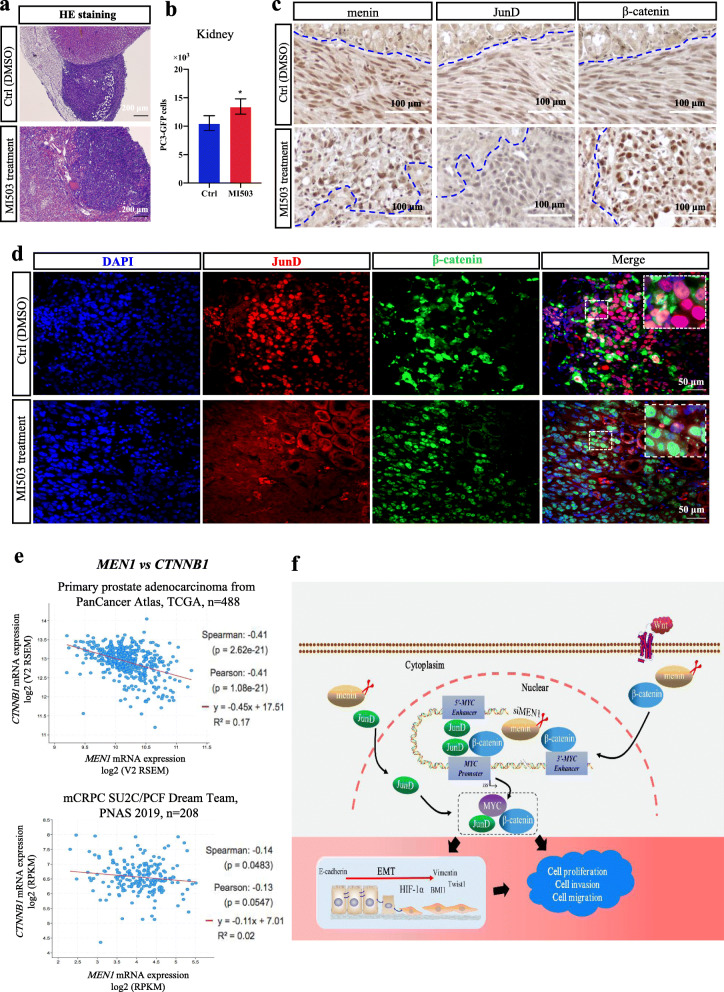
MI503-treated PC3-GFP xenografts display increased tumor growth with nuclear overexpression of β-catenin. **a** Representative images of HE stained xenografts treated with DMSO (upper) and MI503 (lower). Scale bar = 200 μm. **b** qRT-PCR analysis evaluating the number of PC3-GFP cells transplanted in mouse kidney treated (*n* = 11) or not (*n* = 9) with MI503. Representative images of IHC staining (**c,** Scale bar = 100 μm) for menin, JunD and β-catenin or IF staining (**d,** Scale bar = 50 μm) for JunD and β-catenin in xenografts in the Ctrl group (DMSO treatment, upper panels) or MI503 treatment group (lower panels) as indicated. **e** Data mining analyses investigating the clinical correlation between *MEN1* and *CTNNB1* mRNA expression in primary prostate cancer (left panel) and mCRPC (right panel) using existing prostate cancer datasets. **f** Schematic summary of oncosuppressive functions of menin in AR-independent PCa cells. Representative blots of three independent experiments

Importantly, we performed cell line-derived xenografts under mouse kidney capsule using PC3-GFP cells to validate our data in vivo (Fig. S[Media MOESM2]a). Following the transplantation of PC3-GFP cells, mice were treated with either MI503 (*n* = 11) or vehicle (*n* = 9) for 1 month. We observed that MEN1 and JunD inhibition with MI503 gave rise to significantly accelerated tumor growth, compared to the control treatment (Fig. 7a-b). Morphologically, MI503-treated PC3-GFP cells appeared more variable in size and more invasive towards surrounding mouse tissues, with disorientated alignment (Fig. 7a, c, S8b). In agreement with our in vitro observation, qPCR and immunostaining analyses of PC3-GFP xenografts confirmed reduced expression of menin and JunD in xenografted cells, but revealed increased *CTNNB1* transcription and overt nuclear expression of β-catenin in MI503-treated-PC3-GFP cells (Fig.7c-d, S8c-d), suggesting that inhibition of both menin and JunD could still lead to aggravated tumorigenic potential of xenografted PC3-GFP cells, due to activated β-catenin.

In parallel, a significant negative correlation between *MEN1* and *CTNNB1* mRNA expression was seen in two different datasets from TCGA database [35] and mCRPC database [36] (Fig. 7e). These data further support the activated β-catenin due to MI503 treatment observed in PC3-GFP xenografts.

Overall, our data suggest that the activation of both JunD and β-catenin, although they individually play critical roles, is required to produce the effects of menin inactivation in AR-independent PCa cells (Fig. 7f).

## Discussion

In the present study, we uncovered a previously unknown role for menin in preventing AR-independent PCa cells from acquiring an aggravated tumorigenic potential, contrasting drastically with its oncogenic role in AR-dependent PCa cells [23]. More importantly, we demonstrated that *MEN1* silencing in AR-independent PCa cells led to nuclear translocation and, therefore, increased binding of JunD and β-catenin to the regulatory sequences of the *MYC* gene, leading to maintained MYC expression and prominent cellular and molecular alterations.

*MEN1*-KD resulted in an even greater increase in the tumorigenic potential of DU145 and PC3 cells, well-known tumorigenic cell lines, including a marked loss of contact-inhibition, augmented cell anchorage independence and cell migration. Importantly, in vivo xenograft tests in mice further demonstrated that PC3-GFP cells treated with MI503 gave rise to significantly increased tumor growth. In parallel, we noticed that the changes in cellular behavior were accompanied by remarkable alterations in the expression of EMT makers, including BMI1, Twist1, HINF-1α, E-Cadherin and Vimentin. Our data thus suggest that *MEN1* silencing in AR-independent PCa cells affects cell proliferation, cell differentiation and cell migration, resulting in altered cell plasticity and increased tumorigenicity. Both the changes in cell plasticity and abnormal expression of EMT markers are largely documented in PCa, in particular in clinical mCRPC samples [8]. However, we highlighted, for the first time, the involvement of menin in these procedures specifically in AR-independent PCa cells.

JunD was the first menin-interacting partner identified after the identification of the *MEN1* gene, with its possible oncogenic role upon menin inactivation being proposed from the beginning [54]. The hypothesis was further strengthened by menin 3D structure analysis, showing that JunD binds to the same menin protein pocket as KMT2A/B [16]. However, the detailed molecular mechanisms underlying the role of JunD in MEN1 tumors has never been clarified. Interestingly, Wasylishen et al. recently reported that menin plays a tumor suppressive role in mouse Ras-related pancreatic cancer, likely through the activated JunD [55]. It is worth mentioning that our finding is in total agreement with the recent work reporting the role played by JunD in PCa through MYC regulation [8]. Importantly, our data provide new mechanisms showing that *MEN1* silencing led to the prominent nuclear translocation of JunD, which resulted not only in its increased binding to the regulatory sequence of the *MYC* locus, thus maintain MYC expression, but also in the altered expression of EMT markers, contributing to the aggravated tumorigenic potential seen in these *MEN1*-KD AR-independent PCa cells.

The activation of β-catenin and the WNT signaling pathway is considered to be among the most commonly occurring molecular alterations involved in the development and progression of PCa [56, 57]. It has also been suggested that β-catenin could play a critical role in AR-independent CRPC [58]. We and our collaborators have previously demonstrated that menin physically interacts with β-catenin, and that *Men1* deficiency leads to nuclear translocation and activation of the latter in mouse *Men1* insulinoma [43, 44]. Interestingly, we uncovered in the current study that, in AR-independent PCa cells, a similar molecular switch can also occur, accompanied by decreased E-Cadherin and increased Vimentin expression, reminiscent of increased EMT. It is worth mentioning that similar changes were observed in mouse *Men1* insulinomas and mouse *Men1* mammary lesions [59, 60]. Furthermore, our ChIP analyses demonstrated an increase in the binding of β-catenin to the *MYC* promoter, contributing to maintaining MYC expression in these cells. Importantly, the fact that the nuclear expression of β-catenin increased in MI503-treated xenografted PC3-GFP cells further demonstrates that β-catenin activation is critical for aggravated tumorigenicity triggered by menin inactivation observed in the current work.

Notably, we have depicted the increased nuclear expression of several factors related to cell dedifferentiation, including BMI1, Twist1 and HIF-1α. These factors are known to interact with both the JunD and β-catenin pathways and to play relevant roles in cancer progression [61–63]. Based on the published data, we could hypothesize that the activation of JunD and β-catenin may initially lead to the activation of HIF-1α. The latter could subsequently trigger the activation of other factors as previously described [64–67]. The interplay between menin inactivation and the altered expression of EMT makers revealed in the current study may explain the altered cellular activities observed, and it would be relevant to study whether similar situations could occur during PCa progression, especially in the DNPC subtype of mCRPC.

Our study may also provide insight into novel strategies for AR-independent PCa treatment, if these molecular perturbations could be further confirmed in clinical studies. Considering the data obtained from the current work, especially the above mentioned in vivo test, caution should be taken when using inhibitors of menin/MLL interaction, like MI503, in PCa therapeutic assays, especially in mCRPC cases.

## Conclusion

The present work unveiled the activation of the JunD and β-catenin pathways upon menin inactivation specifically in AR-independent cells. Of note, the oncosuppressive role of menin in AR-independent PCa cells is closely associated with maintained MYC expression and altered expression of EMT markers (Fig. 7f), which may pave the way for new strategies for PCa treatment.

## Supplementary Information

**Additional file 1.** Supplementary Materials and Methods.

**Additional file 2.**

## Data Availability

The authors confirm that the data supporting the findings of this study are available within the article [and/or] its supplementary materials.
