# Supplementary material for: MEN1 silencing aggravates tumorigenic potential of AR-independent prostate cancer cells through nuclear translocation and activation of JunD and β-catenin
Source: J Exp Clin Cancer Res. 2021 Aug 26;40:270. doi: 10.1186/s13046-021-02058-7 (PMC8393735; doi:10.1186/s13046-021-02058-7)
Supplement: Supplementary file 1 — Additional file 1. Supplementary Materials and Methods. [file 13046_2021_2058_MOESM1_ESM.docx]

**SUPPLEMENTARY INFOMATION**

***Supplementary Materials and Methods***

*Cell culture and treatment*

LNCaP, 22Rv1 and DU145 were cultured in RPMI medium, PC3 and PC3-AR cells [1] in F-12 medium (Gibco Invitrogen) containing 25 mM glucose and supplemented with 10% (v/v) fetal calf serum (FCS), 2 mM L-Glutamine, 100 unit/mL Penicillin, 100 μg/mL streptomycin, HEPES 10 mM, Sodium Pyruvate 1 mM, at 37°C with 5% CO_2_. Inhibition of the menin-MLL interaction was achieved through the use of MI-503 (Active Biochem), at 1 µM, 2.5 µM, and 5 µM.

*RNA interference and plasmid transfection*

MEN1 siRNA sequences, that are siMEN1(1) (MEN1HSS106462) and siMEN1(3) (MEN1HSS181079), were designed by Invitrogen. *JunD* siRNA (siJunD(1), s7664; siJunD(2), s7665) and *CTNNB1* siRNA (siCTNNB1(1), #146154; siCTNNB1(2), #146154) were purchased from ThermoFisher Scientific. A non-targeting control pool (siCtrl) from Sigma-MERCK was used as a negative control for each RNA interference experiment. Menin overexpressing plasmids (PCI-MEN1) were constructed by our lab in a previous study [2]. For each transfection, 400 ng of the PCI-MEN1 or 400 ng of PCI-neo internal control vector was added to each well. Transfection was performed using Lipofectamine 2000 (Invitrogen) according to the manufacturer’s instructions, and incubated for 72 h.

*Cell proliferation assay*

Cell proliferation assays were performed as described previously [3]. Briefly, cells were seeded onto 96-well culture plates at 5×10^3^ cells for 22Rv1, DU145 or PC3, and at 1×10^4^ cells for LNCaP. 24 h later, cells were treated with 20 nM of siJunD-(1), siJunD-(2) or siJunD-(1)+(2), or 20 nM of siCTNNB1-(1), siCTNNB1-(2) or siCTNNB1-(1)+(2). All experiments were performed in technical and biological triplicates.

*Immunofluorescence (IF) staining*

For IF staining, cells were grown on glass coverslips, then fixed with methanol for 5 min at room temperature. Following fixation, cells were blocked with Dako buffer (S0809, Agilent) for 1 h, and incubated with primary antibodies overnight at 4°C, then with appropriate secondary antibodies conjugated with Alexa 555 (red) or Alexa 488 (green) (Cell Signaling Technology). Cells were counterstained with DAPI (DUO82040, Sigma-Aldrich) for 10 min and visualized by fluorescence microscopy (Eclipse-NiE NIKON microscope).

*Chromatin immunoprecipitation (ChIP) and sequential ChIP (reChIP) assays*

ChIP experiments were performed according to the published protocols with the Millipore ChIP Assay Kit (17-295) [3]. Cells were washed twice and cross-linked with 1% formaldehyde in PBS buffer. The cross-linking reaction was stopped 10 min later by washing with PBS containing glycine (final concentration, 0.1 mol/L). The cells were scraped and lysed. Sonicated chromatin was then immunoprecipitated with antibodies as indicated. Immunoprecipitated fragments were purified using QiaQuick columns (QIAGEN) and used for templates for qPCR. The primers and probes are listed below. The recruitment was presented as the ratio of qPCR result of immunoprecipitated fragments normalized to input. Each qPCR assay was performed in triplicate, and the result was confirmed in three independent experiments.

For reChIP assays [4], the first immunoprecipitated chromatin complexes were washed and eluted with 10 mM dithiothreitol at 37°C for 30 min and diluted 50-fold with ChIP dilution buffer. The second immunoprecipitations were then performed. Briefly, 5 μg of the second antibody was added as indicated, incubated overnight at 4°C, followed by incubation with magnetic streptavidin beads (# 88816, Fisher Scientific). Beads were washed as described above, using a magnet, and the final DNA samples were obtained as described above (ChIP protocol). Experiments were performed in triplicate, and the result was confirmed in three independent experiments.

*Chromosome Conformation Capture (3C) assay and ChIP-3C*

3C assay potentially allows the identification of physical interactions between chromatin segments and is particularly suited to identify chromatin loops formed in genomic regions of up to several hundreds of kilobases in size. 3C was performed as described previously [5, 6, 7] with minor modifications described here. Briefly, 2×10^7^ cells (DU145 or PC3) were fixed with 1% formaldehyde for 10 min at 37°C. Cells were lysed in 10 mM Tris-HCl (pH 8.0), 10 mM NaCl, 0.2% NP-40 and protease inhibitor cocktail. Nuclei were pelleted and chromatin was digested overnight with 2000 U Fat I (NEB, Category Number R0650) and ligated with 4000 U T4 DNA Ligase (NEB) for 4 h at 16°C followed by 30 min at room temperature. 3C samples (ligation or non-ligation) were reverse cross-linked by proteinase K treatment at 65°C for 4 h and purified by phenol/chloroform extraction (Sigma). Typically, 2.5 µg of DNA was recovered at this step, and 100 ng was used for each 3C PCR amplification with specific pairs of primers and resolved on a 2.0% (w/v) agarose gel. The following primers were used for 3C assays: for the ligated fragment (311bp), DP1: 5′-GTGGTTTCCAGTTATCTAAAC-3′ and DP2: 5′-ATAGGGAGGAATGATAGAGGA-3′; for the control fragment (182bp), CP1: 5′-CAAATGCAATGGGAGTTTATT-3′ and CP2: 5′-GAGAGTGGAGGAAAGAAGGGTA-3.

We combined 3C and the ChIP assay to perform the ChIP-3C assay as described previously [8, 9]. For the immunoprecipitation reactions, we digested 50 µg DNA of crosslinked chromatin with Fat I in 500 µL of reaction buffer at 37°C overnight. We added 1% Nonidet P-40 (NP-40) to the remaining sample of digested chromatin and precleared the sample by incubation (1 h at 4°C with rotation) with 50 µL of a 50% suspension of Protein A agarose beads (ChIP assay kit, 17-295, Millipore). Beads were washed as described in the ChIP assay kit, and the final DNA samples were obtained as described above (basic ChIP protocol). Experiments were performed in triplicate, and the result was confirmed in three independent experiments.

***Primers used in the study* (Sequences from 5' to 3')**

| ***For qPCR*** |
| --- |
| huMEN1-F:GACCTGTCCCTCTATCCTCG |
| huMEN1-R:TGACCTCAGCTGTCTGCTCC |
| huMYC-F:AACGATTCCTTCTAACAG |
| huMYC-R:GGCTAAATCTTTC AGTCT |
| huAR-F:CAGTGGATGGGCTGAAAAAT |
| huAR-R:GGAGCTTGGTGAGCTGGTAG |
| huJunD-F:TTTGGAAGAGAGAAGAACAGAG |
| huJunD-R:CCAAGGATTACAAACAGGAATG |
| huCTNNB1-F:TGTGAATCCCAAGTACCAGTGT |
| huCTNNB1-R:CGTCAGACAAAGGAGAAACATT  huHIF1A-F:CTCCATTACCCACCGCTGAA  huHIF1A-R:TCACTGGGACTATTAGGCTCAGGT  huBMI1-F: TGGAAAGTGACTCTGGGAGTGACAAGGC  huBMI1-R:GTCACATGTATTAGCATCTAGAAAGCTGTAATGGC  huTwist1-F:CTAGAGACTCTGGAGCTGGATAACTAAAAA  huTwist1-R:CGACCTCTTGAGAATGCATGCATGAAAAA  huE-cadherin-F:GCCCTGCCAATCCCGATGAAA  huE-cadherin-R:GGGGTCAGTATCAGCCGCT  huVimentin-F:GCTTCAGAGAGAGGAAGCCGAAAA  huVimentin-R:CCGTGAGGTCAGGCTTGGAAA |
| huKi67-F:TGACCCTGATGAGAAAGCTCAA |
| huKi67-R:CCCTGAGCAACACTGTCTTTT |
| huHPRT-F:TATGGCGACCCGCAGCCCT |
| huHPRT-R:CATCTCGAGCAAGACGTTCAG  ***For ChIP-qPCR*** |
| MYC enhancer-F: CATCCAATAAACCTTCCTACCTGA |
| MYC enhancer-R: TGGCAGGTGTCCTAGAGCAT |
| MYC promoter 1-F: GAAGCGTAAATAAAATGTGAAT |
| MYC promoter 1-R: GGCTGCCTTCCAGGCATTAA |
| MYC promoter 2(MYC 5' enhancer)-F:AGGCAACCTCCCTCTCGCCCTA |
| MYC promoter 2(MYC 5' enhancer)-R: AGCAGCAGATACCGCCCCTCCT |
| MYC 3' enhancer-F:GCTCAGTCTTTGCCCCTTTGTGG |
| MYC 3' enhancer-R:TAACACCTTCCCGATTCCCAAGTG |
| Chr 1-F:CGGGGGTCTTTTTGGACCTT |
| Chr 1-R:GAAACACGGCTGCCAGAAAC |
| ***For ChIP-3C-qPCR***  ChIP-3C-qPCR-P1:CATAGTCTAGCTCATTCTGAC |
| ChIP-3C-qPCR-P2:CTATACTTTGCCAATTCTTGG |
| ChIP-3C-qPCR-P3:GTTAATAGATTGGCTATTGAC |
| ChIP-3C-qPCR-P4:CAGCGGCCTGGCAAGGAGAAG |
| ChIP-3C-qPCR-P5:ACGCGCTCTCCAAGTATACGT |
| ChIP-3C-qPCR-P6:CAGAGCGTGGGATGTTAGTGT |
| ChIP-3C-qPCR-P7:GCTCCAGCAGCCTCCCGCGAC |
| ChIP-3C-qPCR-P8:GTTCTCCTCCTCGTCGCATGA |

***Primary antibodies used in the study***

The following primary antibodies were used for Western blot analysis: rabbit anti-menin (1/4,000) (A300-105A, Bethyl), rabbit anti-MYC (1/2,000) (#9402, Cell Signaling Tech.), rabbit anti-AR (1/2,500) (A303-965A, Bethyl), rabbit anti-β-catenin (1/2,000) (#8480S, Cell Signaling Tech.), rabbit anti-JunD (1/2,000)(#720035, Invitrogen), rabbit anti-Vimentin (1/2,000)(#5741S, Cell Signaling Tech.), rabbit anti-E-cadherin (#3195S, Cell Signaling Tech.). Blotting membranes were stripped and reprobed with mouse anti-GAPDH antibody (1/1,000) (sc-47724, Santa Cruz), anti-Na/K-ATPase α1 (1/1,000) (#23565s, Cell Signaling Tech.) or anti-Histone H3 (1/2,000) (#4499, Cell Signaling Tech.) as a loading control.

The primary antibodies used for IF, IHC and ChIP analyses: rabbit anti-menin (1/2,000) (A300-105A, Bethyl), rabbit anti-β-catenin (1/2,000) (#8480S, Cell Signaling Tech.), rabbit anti-JunD (1/2,000) (#720035, Invitrogen), rabbit anti-MYC (1/2,000) (#9402, Cell Signaling Tech.), and goat anti-GFP (1/2,000)(ab5450, Abcam).

***References***

1. Altintas DM, Vlaeminck V, Angelov D, Dimitrov S, Samarut J. Cell cycle regulated expression of NCoR might control cyclic expression of androgen responsive genes in an immortalized prostate cell line. Mol Cell Endocrinol. 2011;332:149–62.

2. Jiang X, Cao Y, Li F, Su Y, Li Y, Peng Y, et al. Targeting β-catenin signaling for therapeutic intervention in *MEN1*-deficient pancreatic neuroendocrine tumours. Nat Commun. 2014;5:5809.

3. Teinturier R, Luo Y, Decaussin-Petrucci M, Vlaeminck-Guillem V, Vacherot F, Firlej V, et al. Men1 disruption in Nkx3.1-deficient mice results in ARlow/CD44+microinvasive carcinoma development with the dysregulated AR pathway. Oncogene. 2021; 40(6):1118-1127.

4. Medeiros RB, Papenfuss KJ, Hoium B, Coley K, Jadrich J, Goh SK, et al. Novel sequential ChIP and simplified basic ChIP protocols for promoter co-occupancy and target gene identification in human embryonic stem cells. BMC Biotechnol. 2009; 9:59.

5. Hagège H, Klous P, Braem C, Splinter E, Dekker J, Cathala G, et al. Quantitative analysis of chromosome conformation capture assays (3C-qPCR). Nat Protoc. 2007;2(7):1722-33.

6. Kodama S, Yamazaki Y, Negishi M. Pregnane X. Receptor Represses HNF4α Gene to Induce Insulin-Like Growth Factor-Binding Protein IGFBP1 that Alters Morphology of and Migrates HepG2 Cells. Mol Pharmacol. 2015; 88(4): 746-57.

7. Hagège H, Klous P, Braem C, Splinter E, Dekker J, Cathala G, et al. Quantitative analysis of chromosome conformation capture assays (3C-qPCR). Nat Protoc. 2007;2(7):1722-33. doi: 10.1038/nprot.2007.243. PMID: 17641637.

8. Cai S, Lee CC, Kohwi-Shigematsu T. SATB1 packages densely looped, transcriptionally active chromatin for coordinated expression of cytokine genes. Nat Genet. 2006; 38(11): 1278-88.

9. Sancho A, Li S, Paul T, Zhang F, Aguilo F, Vashisht A, et al. CHD6 regulates the topological arrangement of the CFTR locus. Hum Mol Genet. 2015; 24(10): 2724-32.

***Legends for supplementary figures***

***Fig. S1 MEN1 silencing reduces cell growth and cell migration of AR-dependent PCa cells and PC3-AR cells.* a** Representative images of foci formation assays and their quantification in *MEN1*-KD LNCaP, 22Rv1 and PC3-AR cells. **b** Western blot analysis showing the efficacy of siMEN1(1)+(3) in LNCaP, 22Rv1 and PC3-AR cells, related to Fig. 1a-b. **c** Representative images of wound healing assays and their quantification using siMEN1- or siCtrl-transfected 22Rv1 and PC3-AR cells. Graphs showing cell migration displayed in terms of the % wound closure 12 h post-wounding (t = 0, as control). Scale bar = 200 µm. **d** Quantitative RT-PCR (qRT-PCR) analysis of *MYC* transcripts (left panel) and Western blot analysis of MYC protein levels (right panle) in PCa cells treated with siCtrl or siMEN1(1)+(3) for 72 h. Representative blots of three independent experiments.

***Fig. S2 MEN1 silencing triggers the nuclear translocation of JunD in AR-independent PCa cells.*** **a** qRT-PCR analysis of *JunD* and *MYC* mRNA expression in LNCaP and PC3 cells treated with MI503 (2.5 µM). **b** qRT-PCR analysis of *JunD* mRNA expression in *MEN1*-KD LNCaP and PC3 cells. **c** Western blot analysis of JunD in *MEN1*-KD LNCaP and PC3 cells. **d** Incucyte ZOOM analysis of DU145 and PC3 cell proliferation upon transfection with siCtrl, siJunD(1), siJunD(2) or siJunD(1)+(2). **e** Double IF staining for menin and JunD expression in siCtrl- or siMEN1(1)+(3)-transfected PC3 cells. Scale bar = 25 µm. **f** qRT-PCR analysis of *MEN1* mRNA expression in siCtrl- or siJunD(1)+(2)-transfected DU145 and PC3 cells. **g** Western blot analysis of JunD in *JunD*-KD DU145 and PC3 cells. Representative blots of three independent experiments.

***Fig. S3 MEN1 silencing elevates JunD binding to the MYC locus in AR-independent PCa cells.*** **a** 3C detection results (DP1-DP2 fragment, right panel) and control (CP1-CP2, left panel) by PCR in siCtrl or siMEN1(1)+(3)-transfected PC3 cells upon ligation or non-ligation. ChIP-3C qPCR analysis showing menin (**b**) and JunD (**c**) binding to the MYC 5’ enhancer (P3-P4), promoter (P5-P6) and looping fragment (P3-P6), in PC3 cells transfected with siCtrl or siMEN1(1)+(3) under ligation or non-ligation conditions. P1-P2 and P7-P8 served as negative controls.

***Fig. S4 MEN1 silencing induces the nuclear translocation of β-catenin in AR-independent, not AR-dependent, PCa cells.*** **a** Double IF staining showing menin and β-catenin in siCtrl- or siMEN1(1)+(3)-transfected PC3 cells. Scale bar = 25 µm. **b** qRT-PCR analysis for mRNA expression of *CTNNB1* in *MEN1*-KD LNCaP and 22Rv1 cells. **c** Western blot showing β-catenin protein levels in different subcellular fractions in siCtrl- or siMEN1(1)+(3)-transfected LNCaP cells as indicated. **d** Double IF staining for menin and β-catenin expression in siCtrl- or siMEN1(1)+(3)-transfected LNCaP cells. Scale bar = 25 µm. **e** Quantitative RT-PCR (qRT-PCR) analysis of *MEN1* transcripts (left panel) and Western blot analysis of menin protein levels (right panel) in DU145 and PC3 cells treated with siCtrl or siCTNNB1(1)+(2) for 72 h. Representative blots of three independent experiments. **f** Incucyte ZOOM analysis showing proliferation of DU145 and PC3 cells upon transfection with siCtrl, siCTNNB1(1), siCTNNB1(2) or siCTNNB1(1)+(2).

***Fig. S5 β-catenin replaces menin to bind to the MYC promoter in MEN1-KD AR-independent Pa cells.*** ChIP-qPCR analysis to assess β-catenin (**a**) and menin (**b**) binding to the *MYC* promoter and the *MYC* 3’enhancer in siCTNNB1(1)+(2)- or siMEN1(1)+(3)-treated PC3 cells. **c** ChIP-reChIP analysis evaluating the effect of *MEN1*-KD on menin (left panel) and β-catenin (right panel) co-occupancy on the *MYC* promoter in PC3 cells. **d** ChIP-qPCR analysis evaluating the effect of *MEN1*-KD on the binding of β-catenin to the *MYC* 3’enhancer in DU145 and PC3 cells treated with siCrtl or siMEN1(1)+(3). **e** ChIP-reChIP analysis assessing the effects of siCTNNB1(1)+(2) on menin (left panel) and β-catenin (right panel) co-occupancy on the *MYC* promoter in PC3 cells as indicated.

***Fig. S6 Both JunD and β-catenin are needed for increased tumorigenic potential triggered by menin inactivation in AR-independent PCa cells.*** Foci formation for PC3 (**a**) or Incucyte ZOOM (**b**) analysis of DU145 and PC3 cell growth upon transfection with siMEN1, siJunD, siCTNNB1, siMEN1+siJunD, siMEN1+ siCTNNB1 or siMEN1+siJunD+siCTNNB1.

***Fig. S7 Menin overexpression reduces cell proliferation and migration in AR-independent PCa cells.* a** Representative images of foci formation assays and their quantification in PCI-neo or PCI-*MEN1*-transfected DU145 and PC3 cells. **b** Representative images of wound healing assays and their quantification using PCI-neo or PCI-*MEN1*-transfected DU145 and PC3 cells. Graphs showing cell migration displayed in terms of the % wound closure 12 h post-wounding (t = 0, as control). Scale bar = 200 µm.

***Fig. S8 MI503-treated xenografted PC3-GFP cells displayed accelerated tumor growth with increased nuclear β-catenin expression.*** **a** Schematic diagram of the strategy used for evaluating the effect of MI503 on PC3-GFP cell growth by xenografts in mice. **b** Representative images of IHC staining for GFP in xenografts from the control group (Ctrl: DMSO treatment, upper panel) or MI503 treatment group (lower panel). **c** qRT-PCR analysis detecting *MEN1*, *JunD* and *CTNNB1* mRNA expression in DMSO- or MI503-treated xenografts. **d** Representative images of IF staining for menin and GFP in xenografts from the control group or MI503 treatment group (lower panels) as indicated.
