# Supplementary figures and images for: MEN1 silencing aggravates tumorigenic potential of AR-independent prostate cancer cells through nuclear translocation and activation of JunD and β-catenin
Source: J Exp Clin Cancer Res. 2021 Aug 26;40:270. doi: 10.1186/s13046-021-02058-7 (PMC8393735; doi:10.1186/s13046-021-02058-7)

Fig. S1

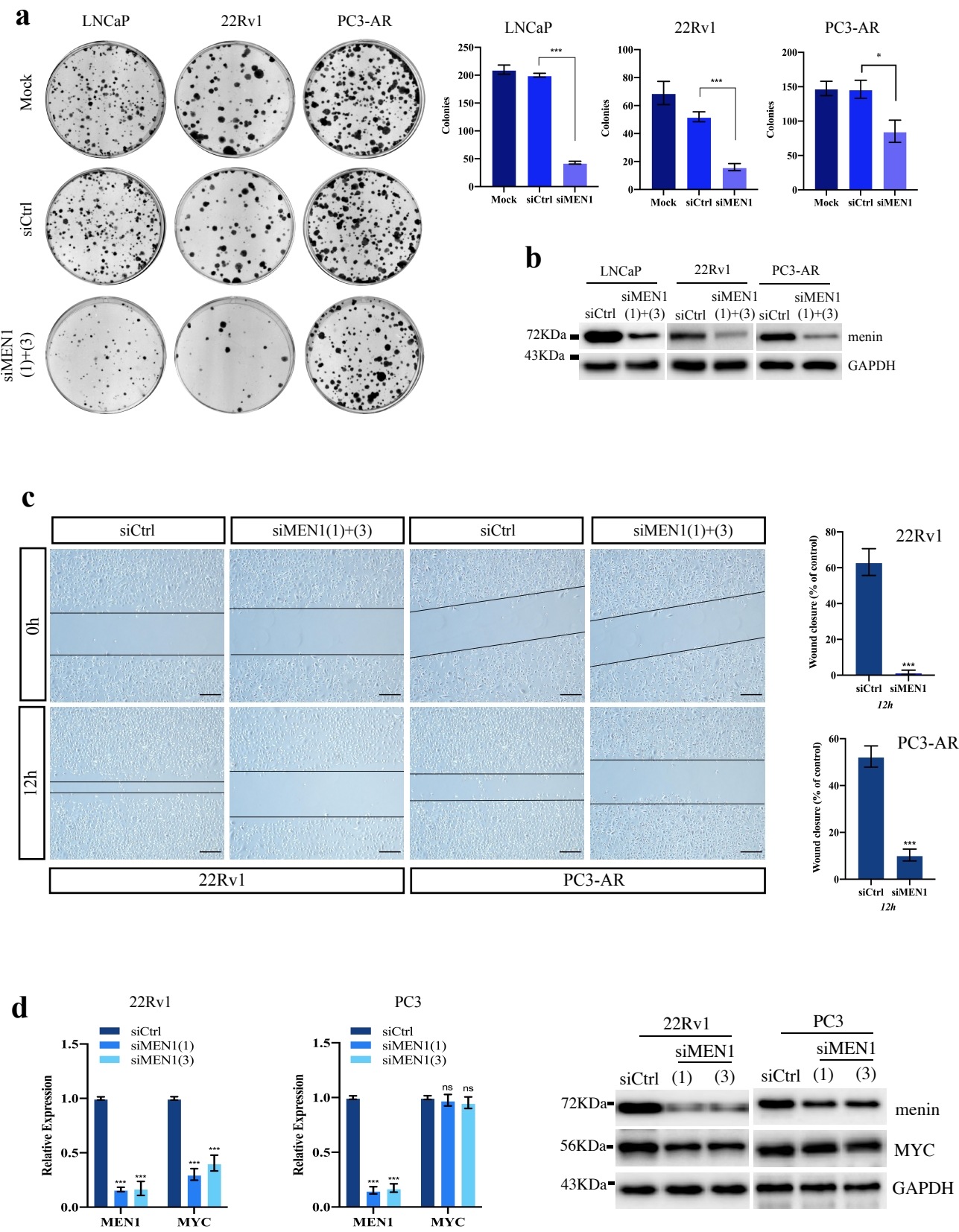

Fig. S2

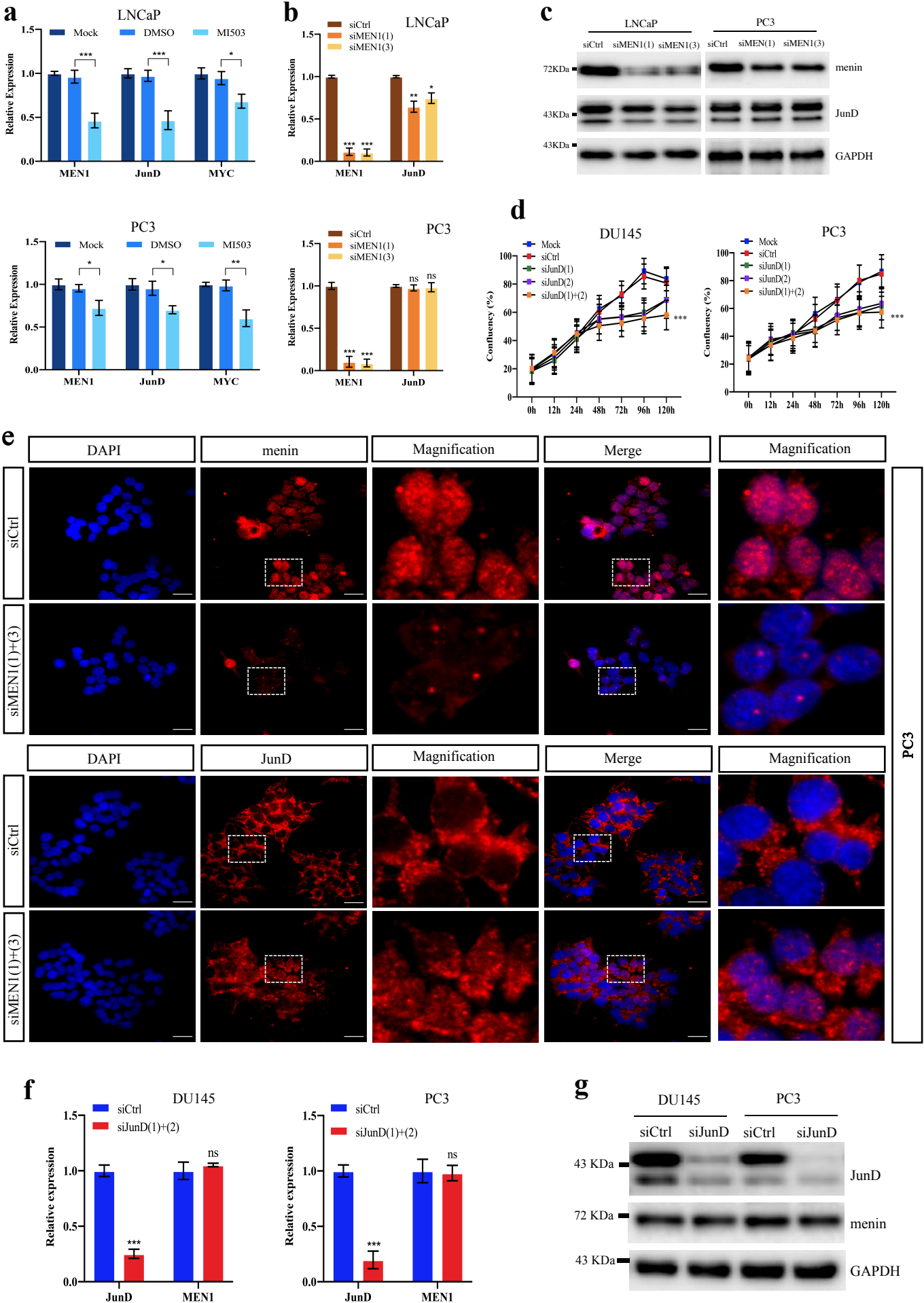

Fig. S3

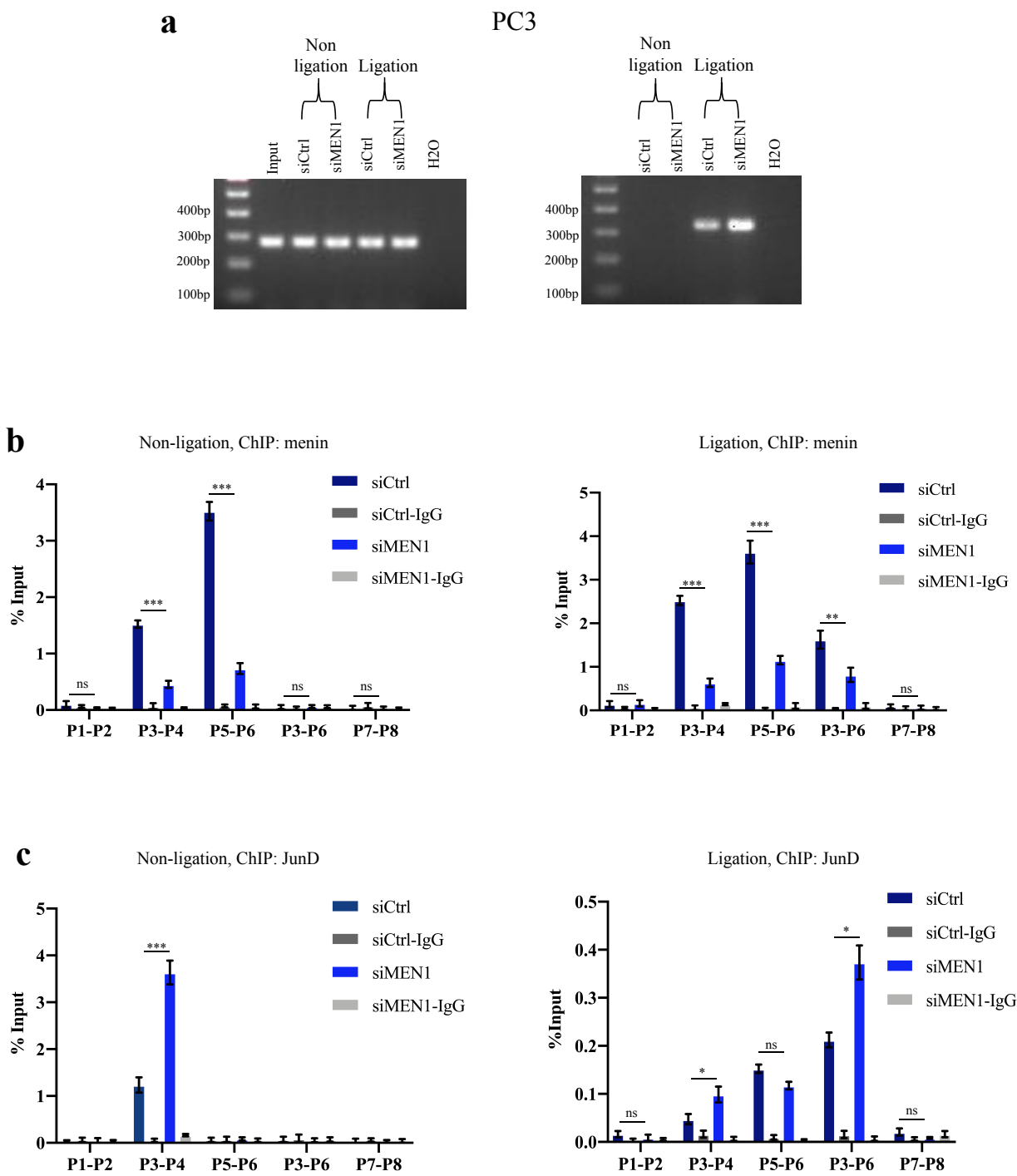

Fig. S4

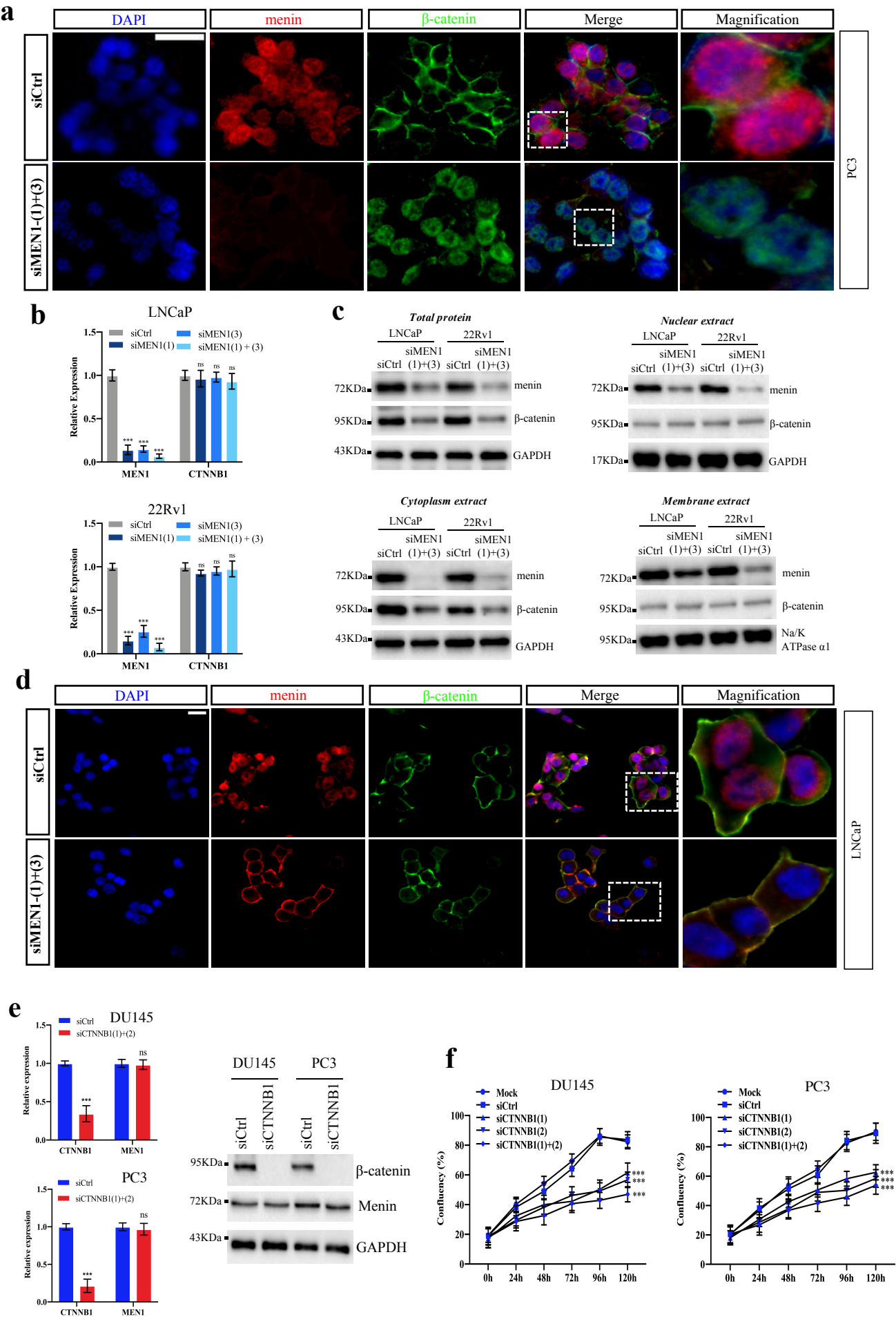

Fig. S5

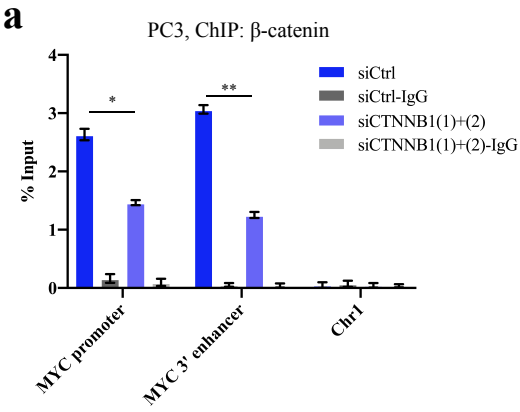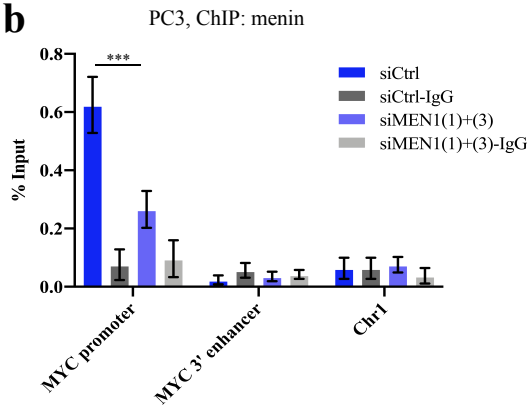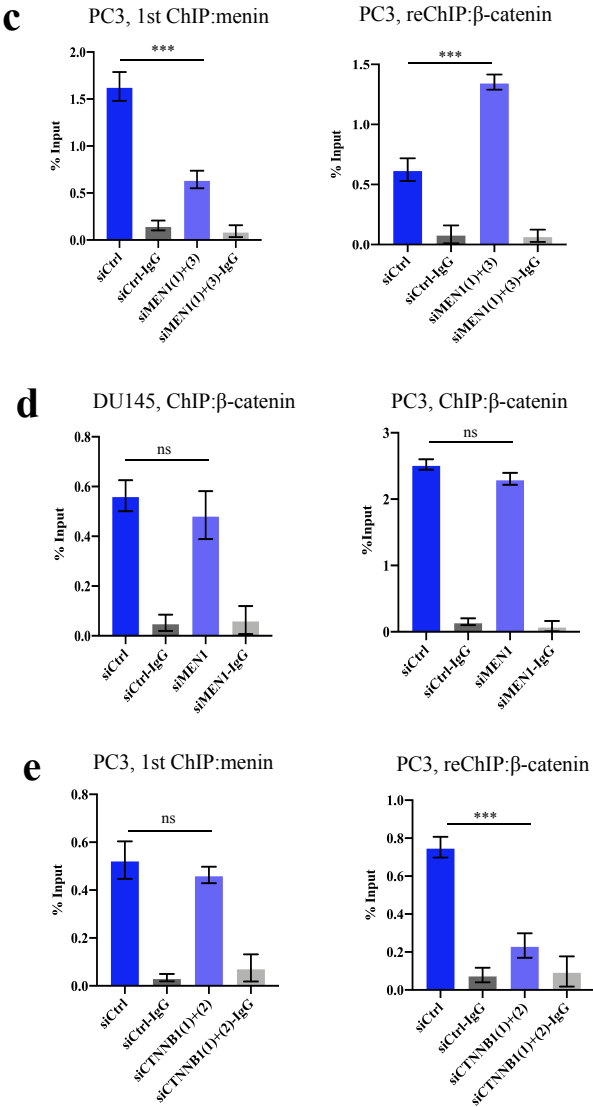

Fig. S6

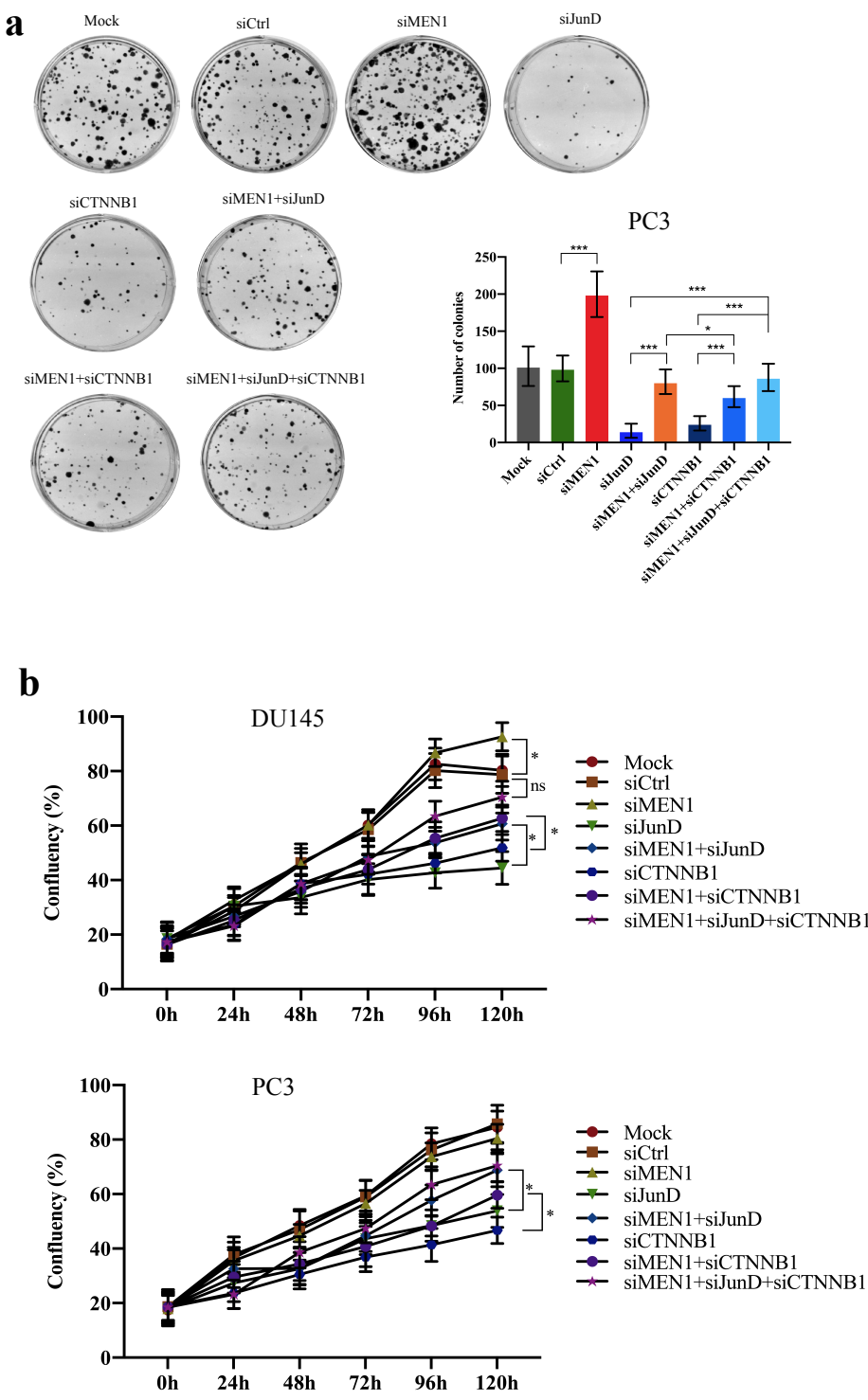

Fig. S7

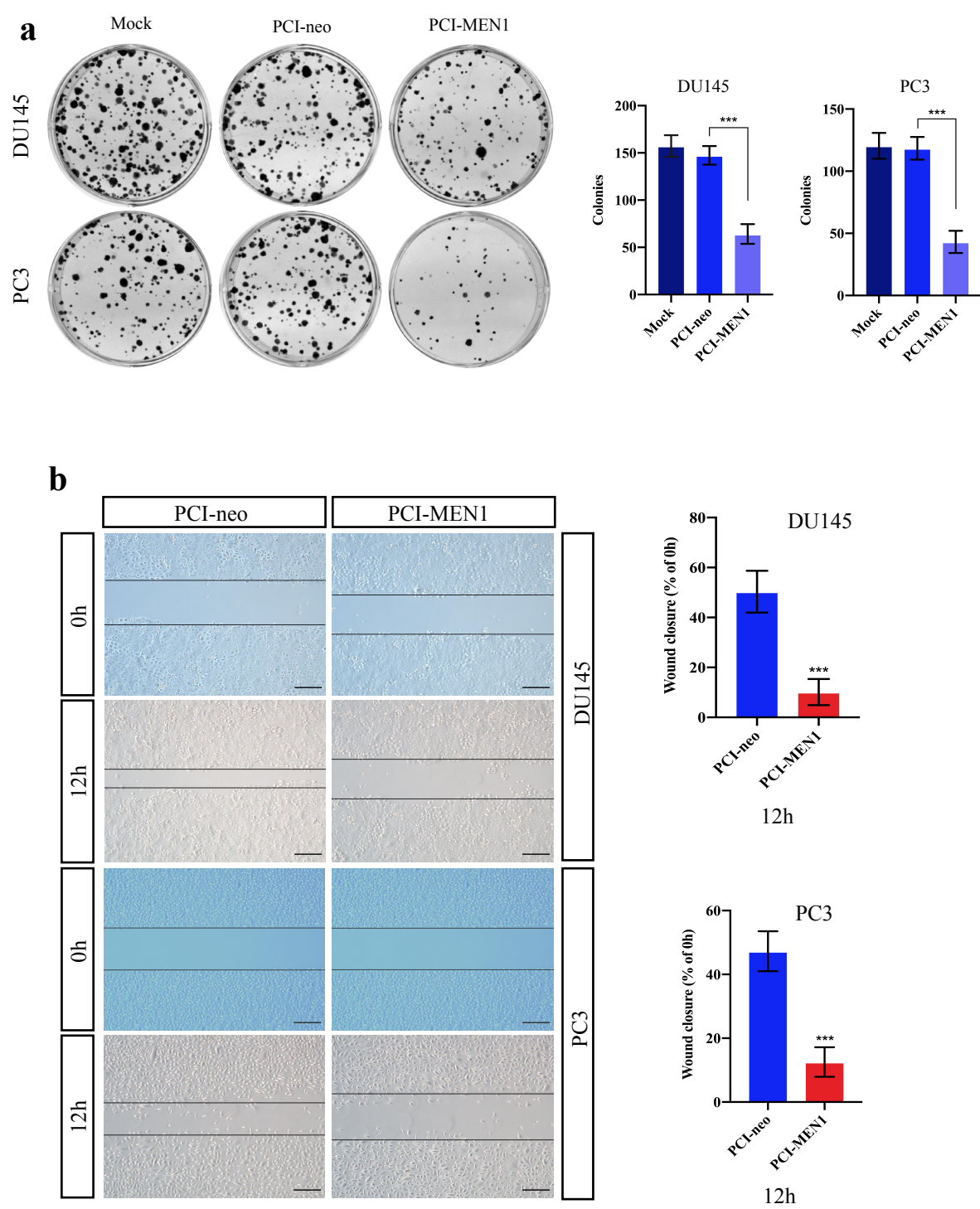

Fig. S8

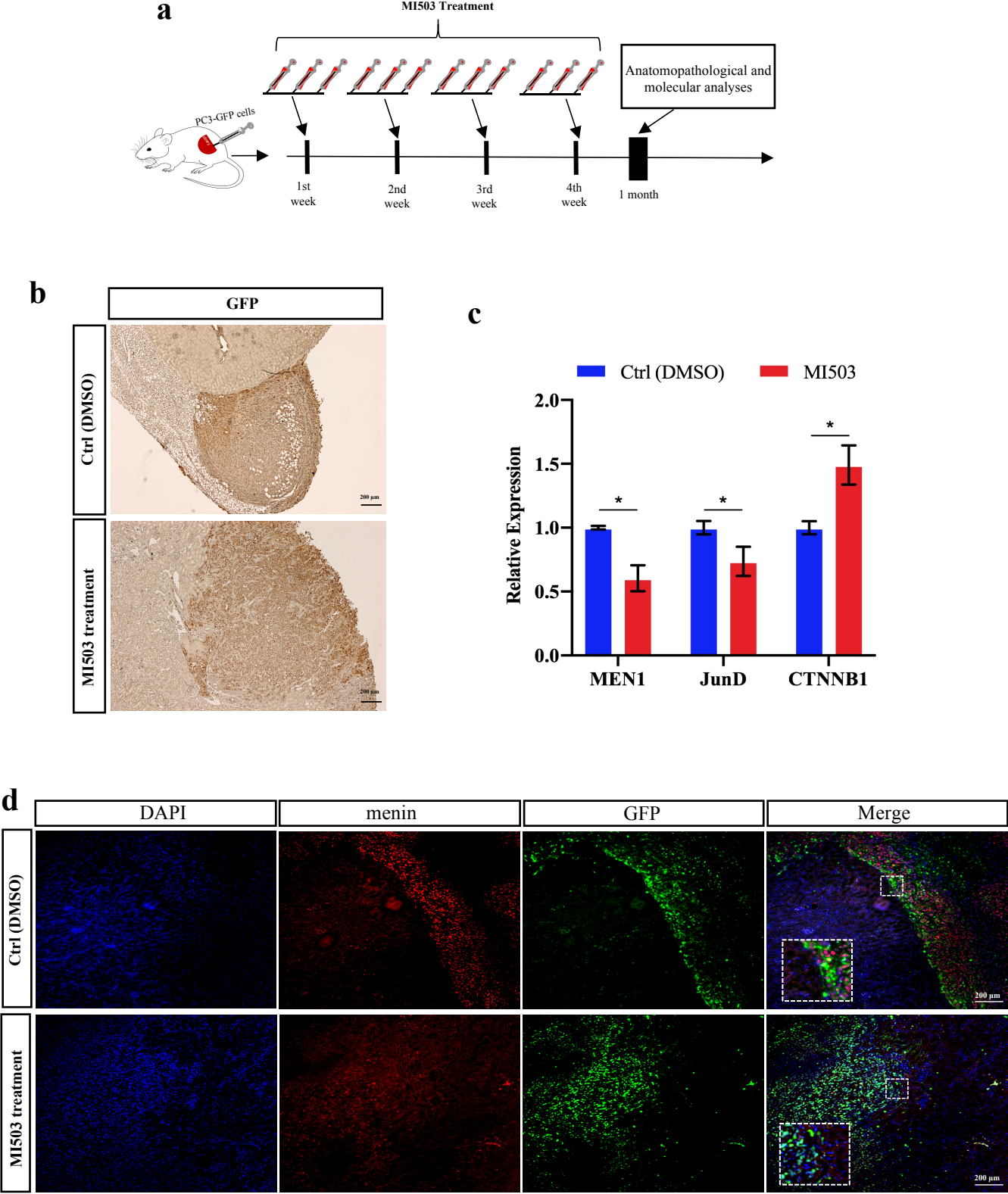

Supplement: Supplementary file 2 — Additional file 2. [file 13046_2021_2058_MOESM2_ESM.pdf]
